# Supplementary figures and images for: Genomic changes in Kaposi Sarcoma-associated Herpesvirus and their clinical correlates
Source: PLoS Pathog. 2022 Nov 28;18(11):e1010524. doi: 10.1371/journal.ppat.1010524 (PMC9731496; doi:10.1371/journal.ppat.1010524)

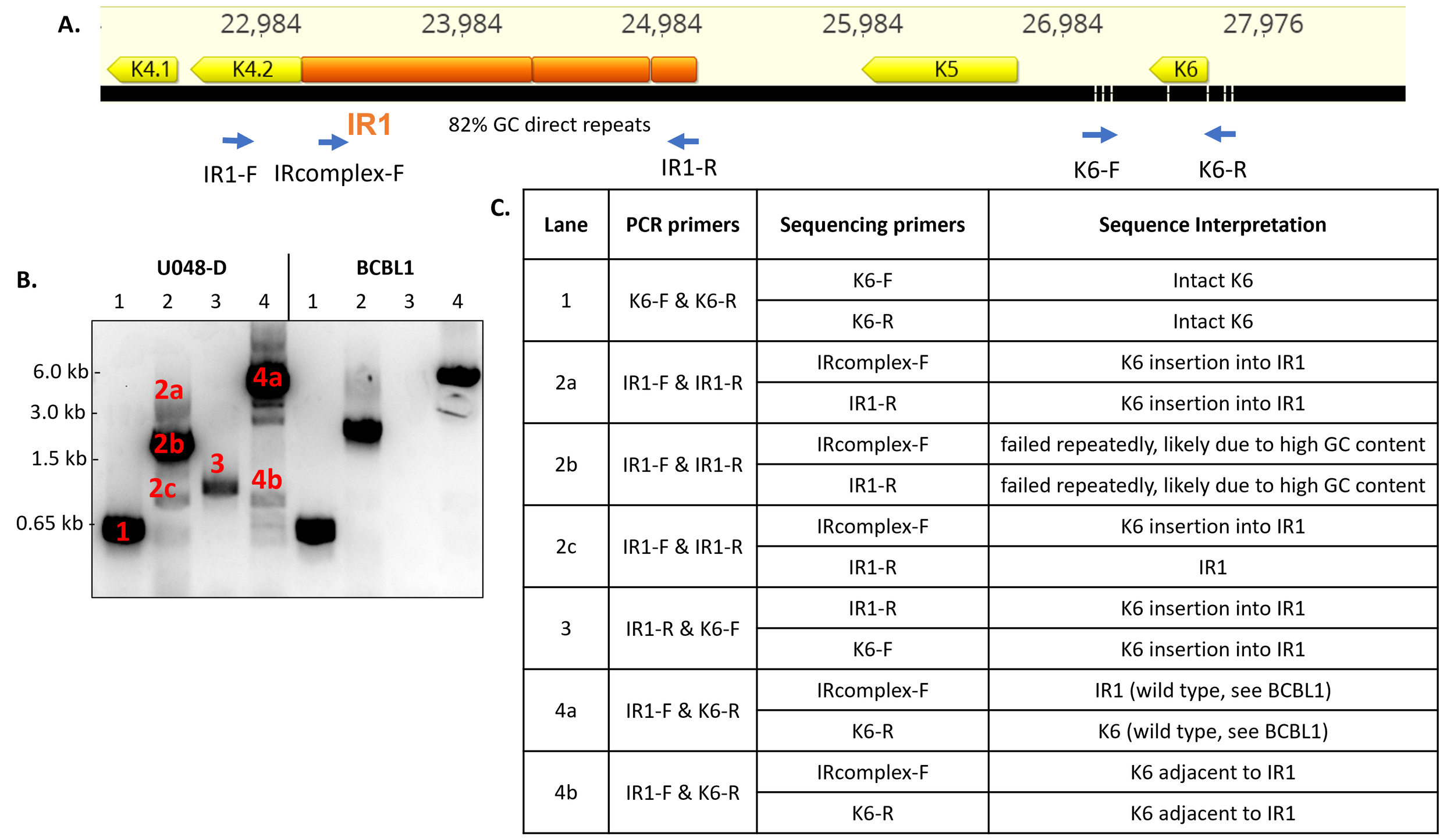

Supplement: S1 Fig — A. Primers (blue arrows) used to PCR across breakpoint junctions in the tumor DNA extract is shown below a KSHV genome section (GK18 numbering) from K4.2 through IR1 and K6. ORFs are in yellow, repetitive sequences in orange. B. PCR products from indicated primers separated on a 0.8% agarose gel. Visible bands were extracted and sequenced. DNA from the KSHV-infected BCBL1 cell line was used as control. C. Results from sequencing the numbered bands in B. Sequencing of bands 2a and 2c using IR1 primers from both ends unexpectedly show reads mapping to K6 sequences. Sequencing for band 2b failed repeatedly, perhaps due to GC content. Bands 3 and 4b, which are not present in BCBL1, show that K6 sequences are connected directly to IR1 sequences at ~1 kb or less, suggesting an insertion. Band 4a shows the normal expected size between IRcomplex-F and K6-R, as seen in BCBL1. (TIF) [file ppat.1010524.s001.tif]

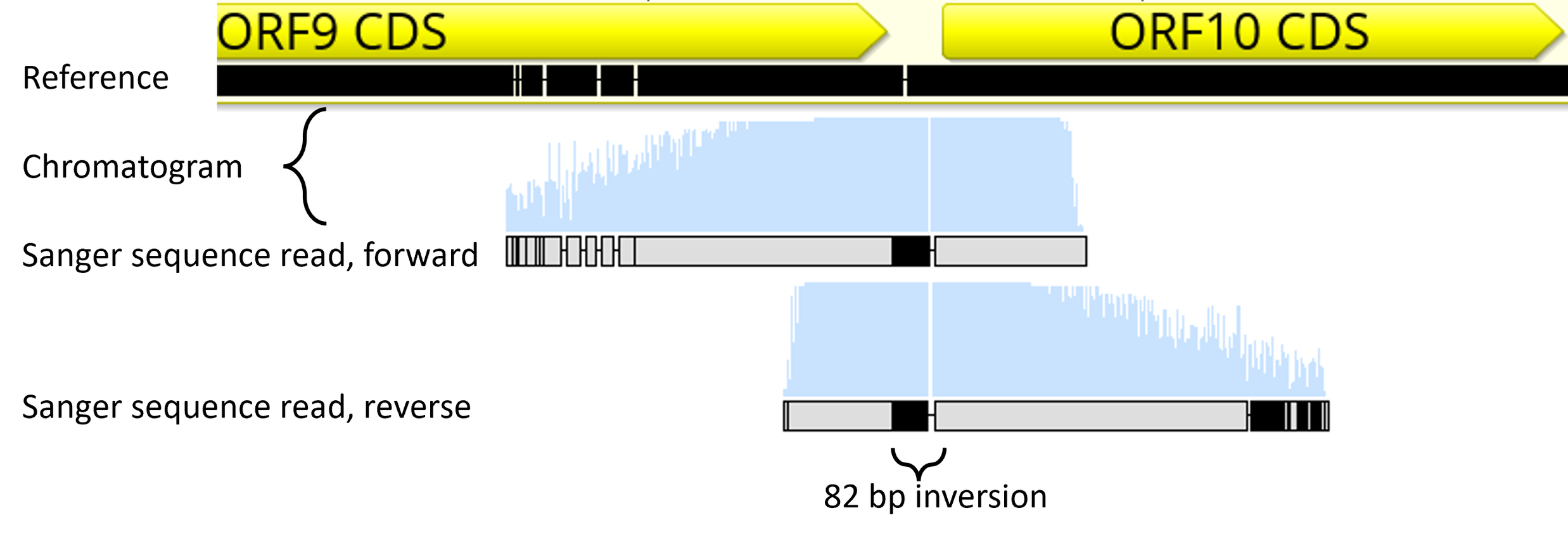

Supplement: S2 Fig — An 82 bp inversion was detected from coordinates 14,481 to 14,562. Below are Sanger sequence reads aligned to reference. Black marks represent mismatches to the reference, and the height of the chromatogram indicates the quality of the read. The gap next to the inversion represents an 8 bp deletion. (TIF) [file ppat.1010524.s002.tif]

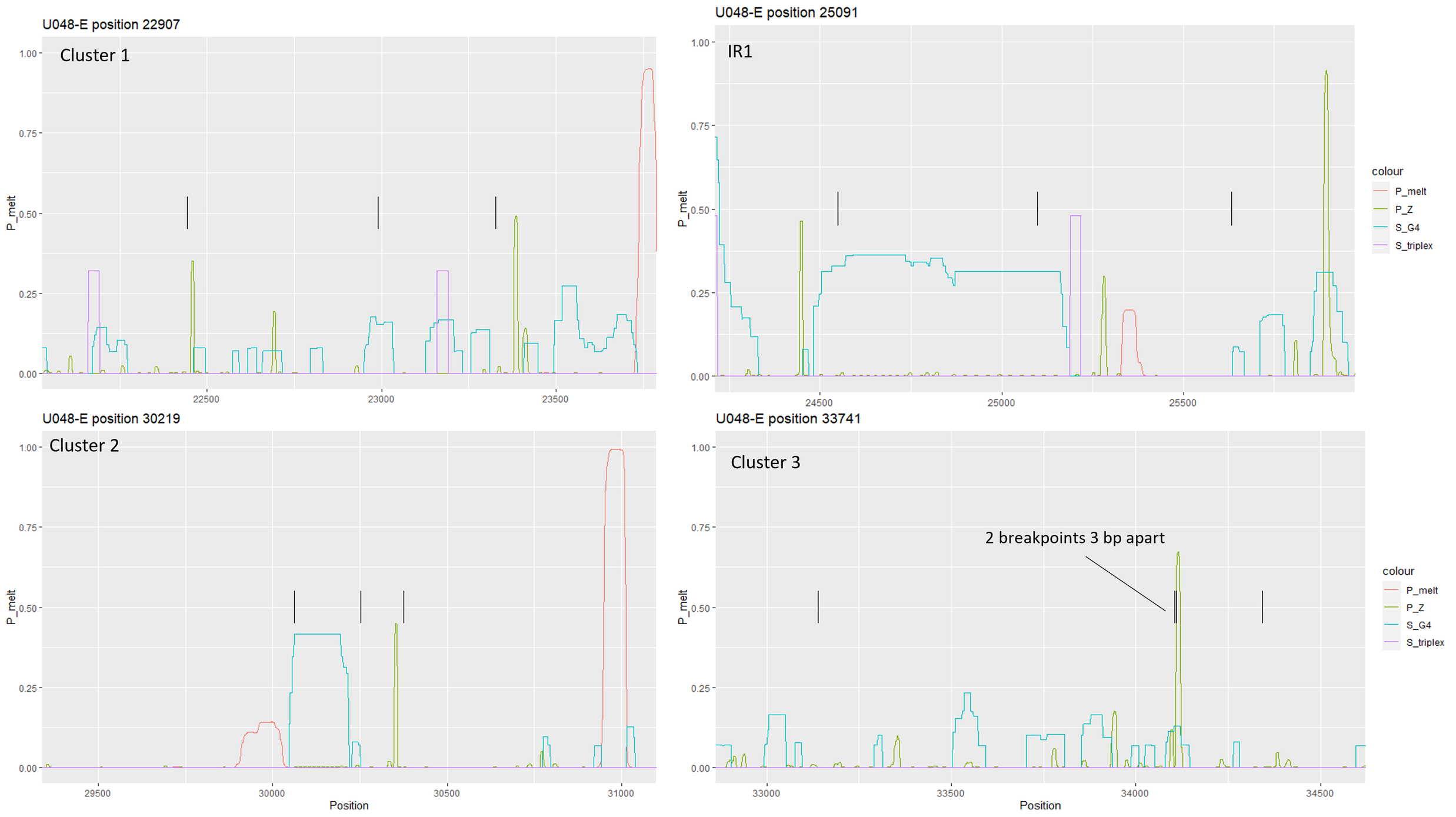

Supplement: S3 Fig — Cruciform, local melt region, Z-DNA, cruciform, G-quadruplex and triplex DNA at individual clusters of breakpoints are shown. No cruciform was found. G-quadruplex scores are normalized to 300, near the human genome maximum (https://pqsfinder.fi.muni.cz/genomes), and triplex scores are normalized to a maximum of 50. (TIF) [file ppat.1010524.s003.tif]

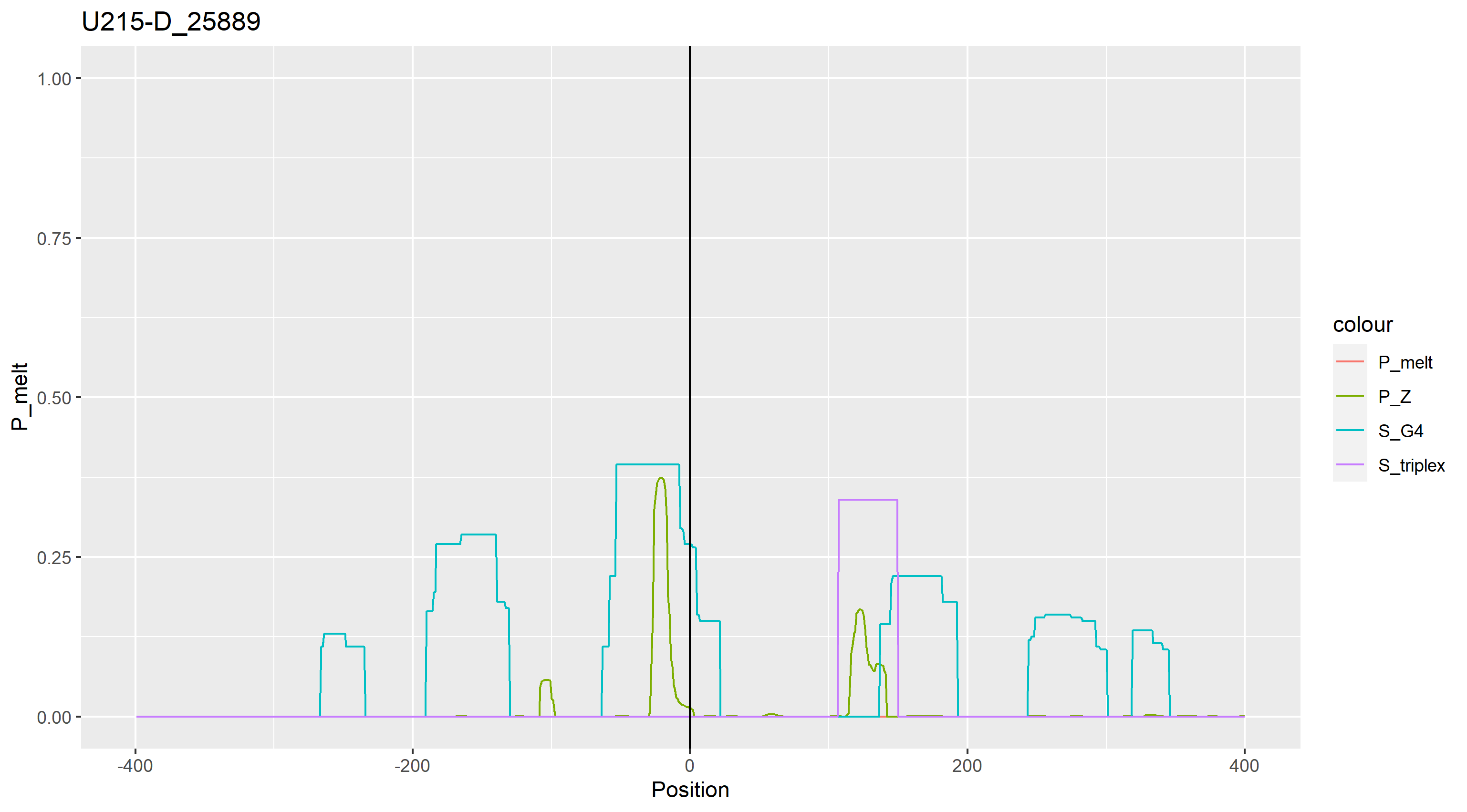

Supplement: S1 Graphs — The coordinate numbers in the figures of some breakpoints differ slightly from those in Column D of S2 Table because working draft genomes were used for non-B-DNA analyses, while S2 Table lists coordinates in the finished genomes uploaded to Genbank. The coordinate differences come from refinements in the genome termini and repeat regions, outside the analyzed sequences. (ZIP) [file ppat.1010524.s014.zip › Supplementary graphs/U215-D_25889.png]

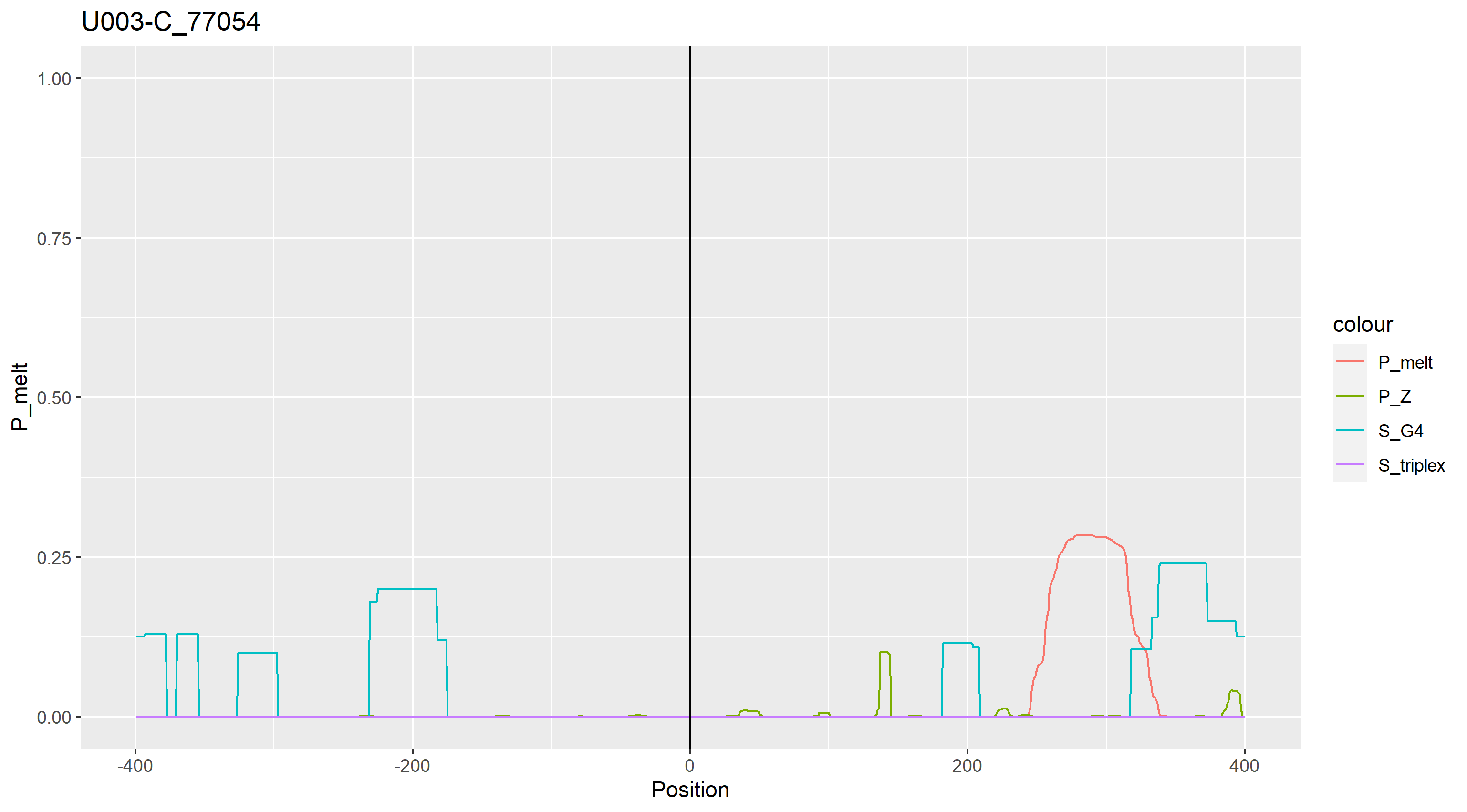

Supplement: S1 Graphs — The coordinate numbers in the figures of some breakpoints differ slightly from those in Column D of S2 Table because working draft genomes were used for non-B-DNA analyses, while S2 Table lists coordinates in the finished genomes uploaded to Genbank. The coordinate differences come from refinements in the genome termini and repeat regions, outside the analyzed sequences. (ZIP) [file ppat.1010524.s014.zip › Supplementary graphs/U003-C_77054.png]

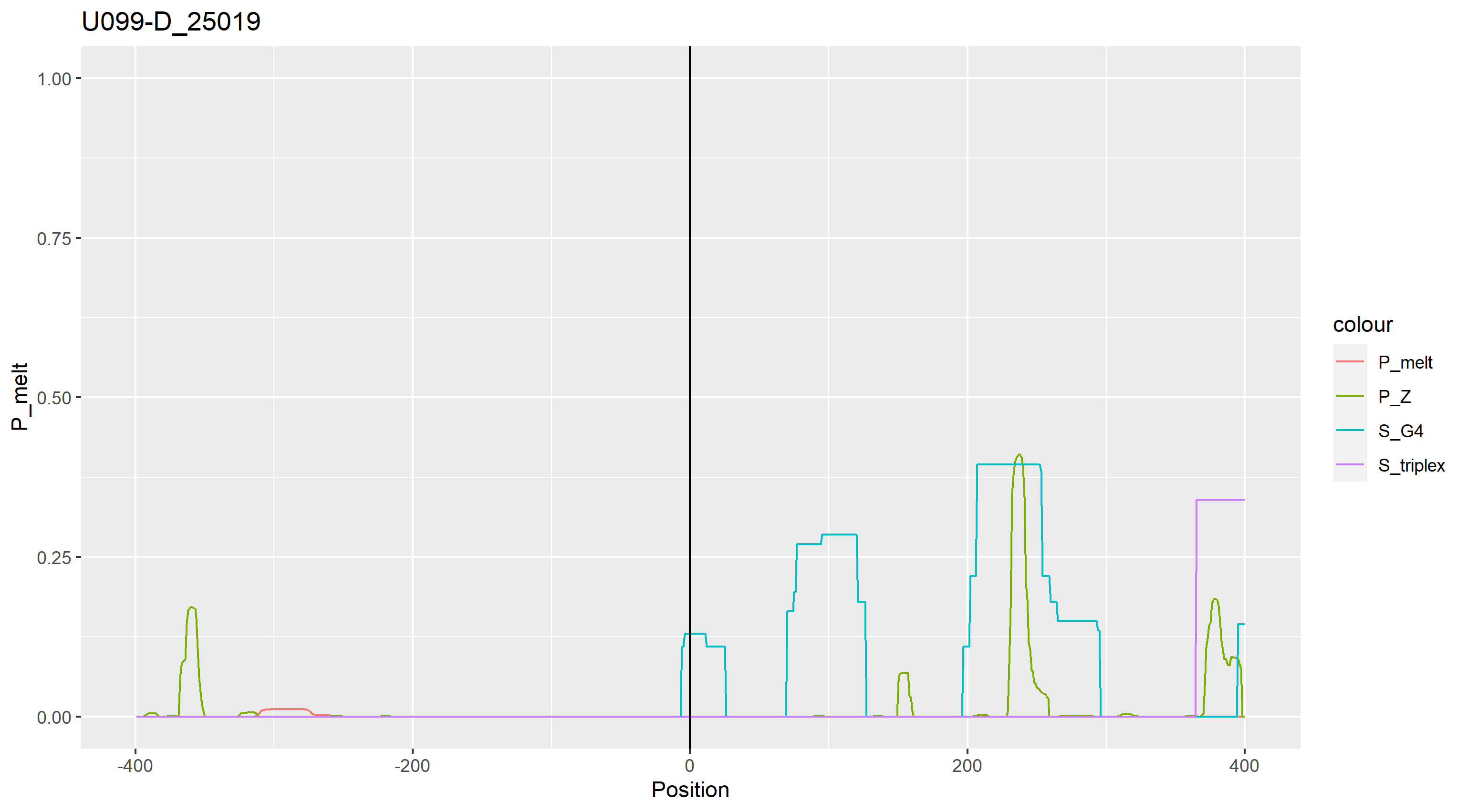

Supplement: S1 Graphs — The coordinate numbers in the figures of some breakpoints differ slightly from those in Column D of S2 Table because working draft genomes were used for non-B-DNA analyses, while S2 Table lists coordinates in the finished genomes uploaded to Genbank. The coordinate differences come from refinements in the genome termini and repeat regions, outside the analyzed sequences. (ZIP) [file ppat.1010524.s014.zip › Supplementary graphs/U099-D_25019.png]

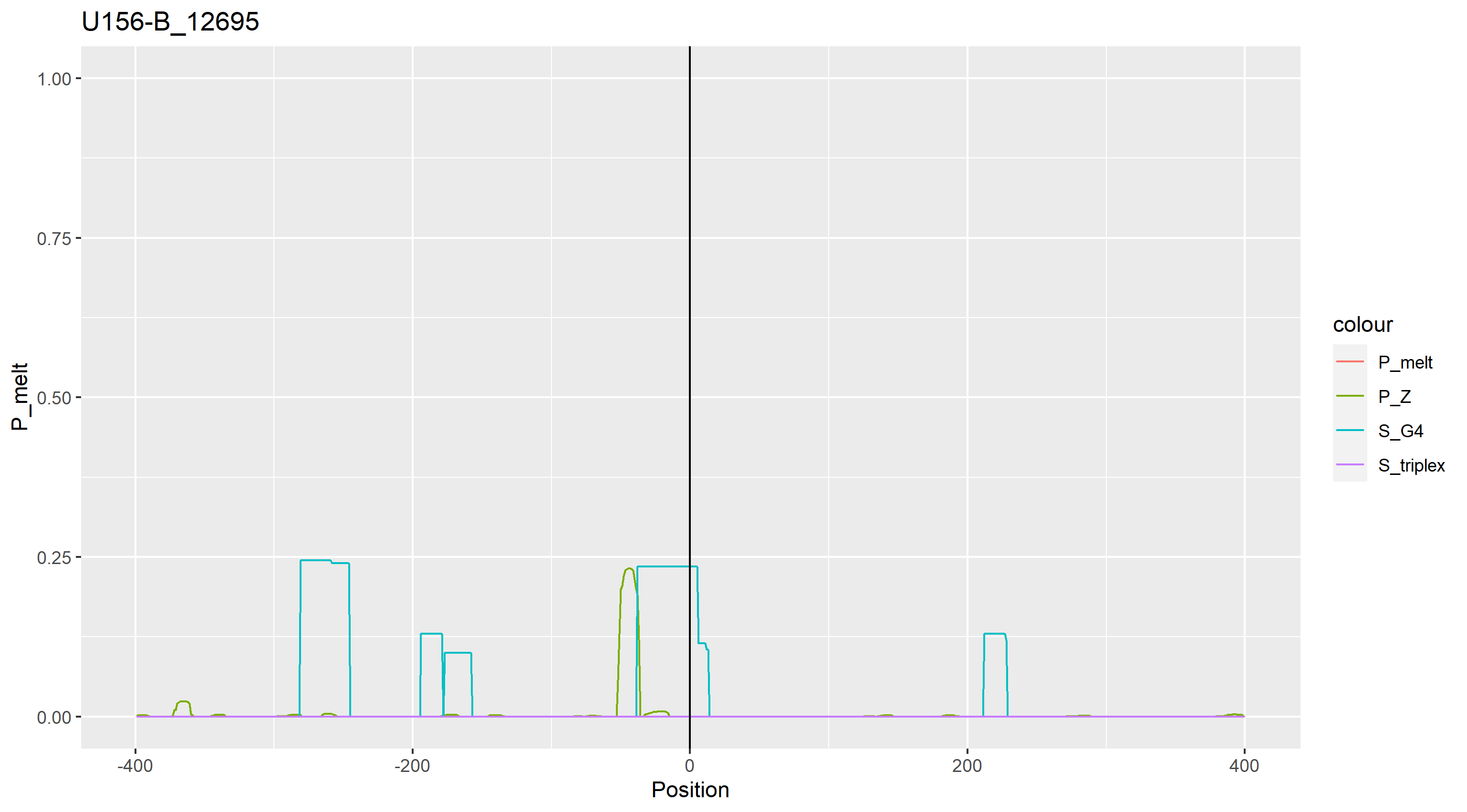

Supplement: S1 Graphs — The coordinate numbers in the figures of some breakpoints differ slightly from those in Column D of S2 Table because working draft genomes were used for non-B-DNA analyses, while S2 Table lists coordinates in the finished genomes uploaded to Genbank. The coordinate differences come from refinements in the genome termini and repeat regions, outside the analyzed sequences. (ZIP) [file ppat.1010524.s014.zip › Supplementary graphs/U156-B_12695.png]

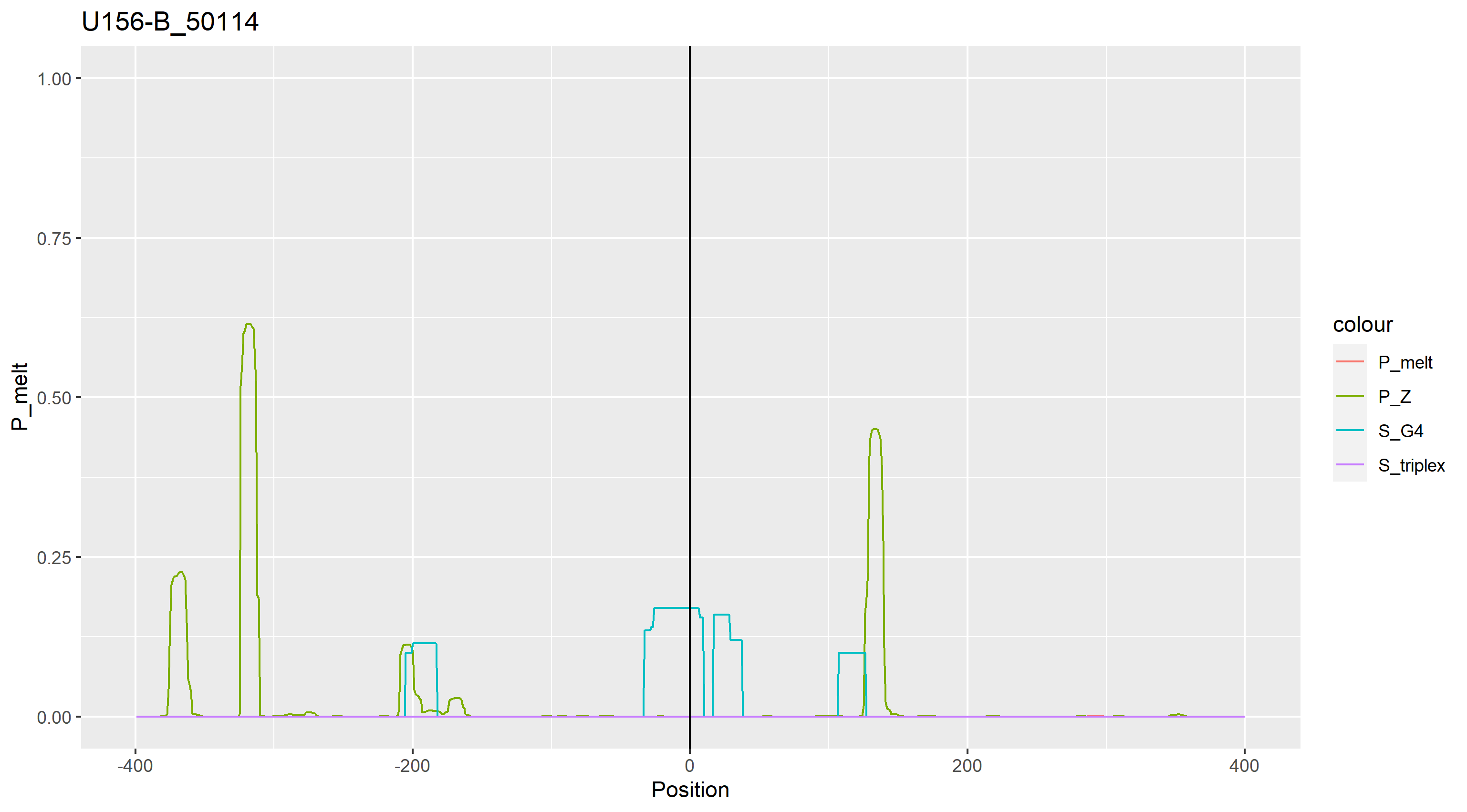

Supplement: S1 Graphs — The coordinate numbers in the figures of some breakpoints differ slightly from those in Column D of S2 Table because working draft genomes were used for non-B-DNA analyses, while S2 Table lists coordinates in the finished genomes uploaded to Genbank. The coordinate differences come from refinements in the genome termini and repeat regions, outside the analyzed sequences. (ZIP) [file ppat.1010524.s014.zip › Supplementary graphs/U156-B_50114.png]

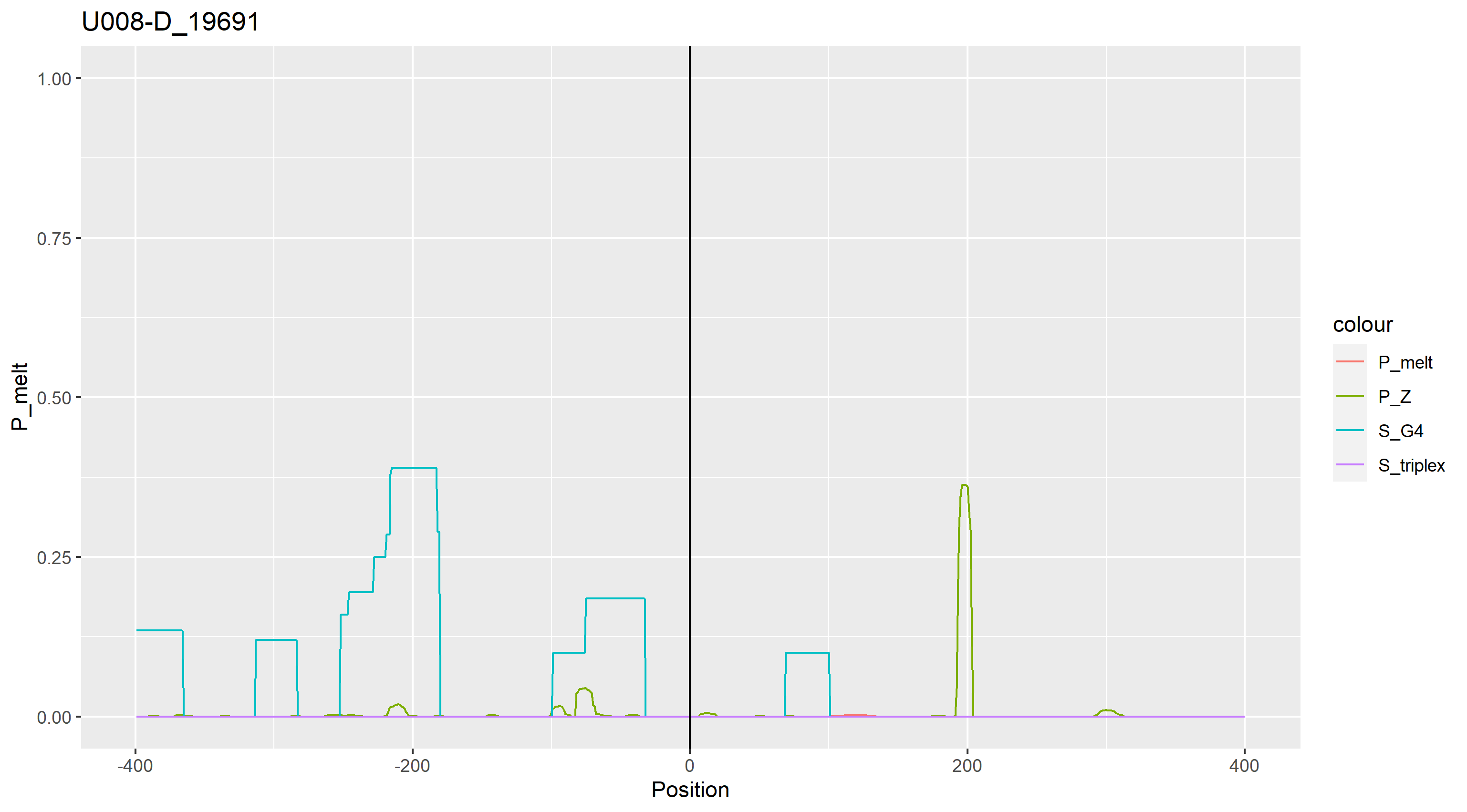

Supplement: S1 Graphs — The coordinate numbers in the figures of some breakpoints differ slightly from those in Column D of S2 Table because working draft genomes were used for non-B-DNA analyses, while S2 Table lists coordinates in the finished genomes uploaded to Genbank. The coordinate differences come from refinements in the genome termini and repeat regions, outside the analyzed sequences. (ZIP) [file ppat.1010524.s014.zip › Supplementary graphs/U008-D_19691.png]

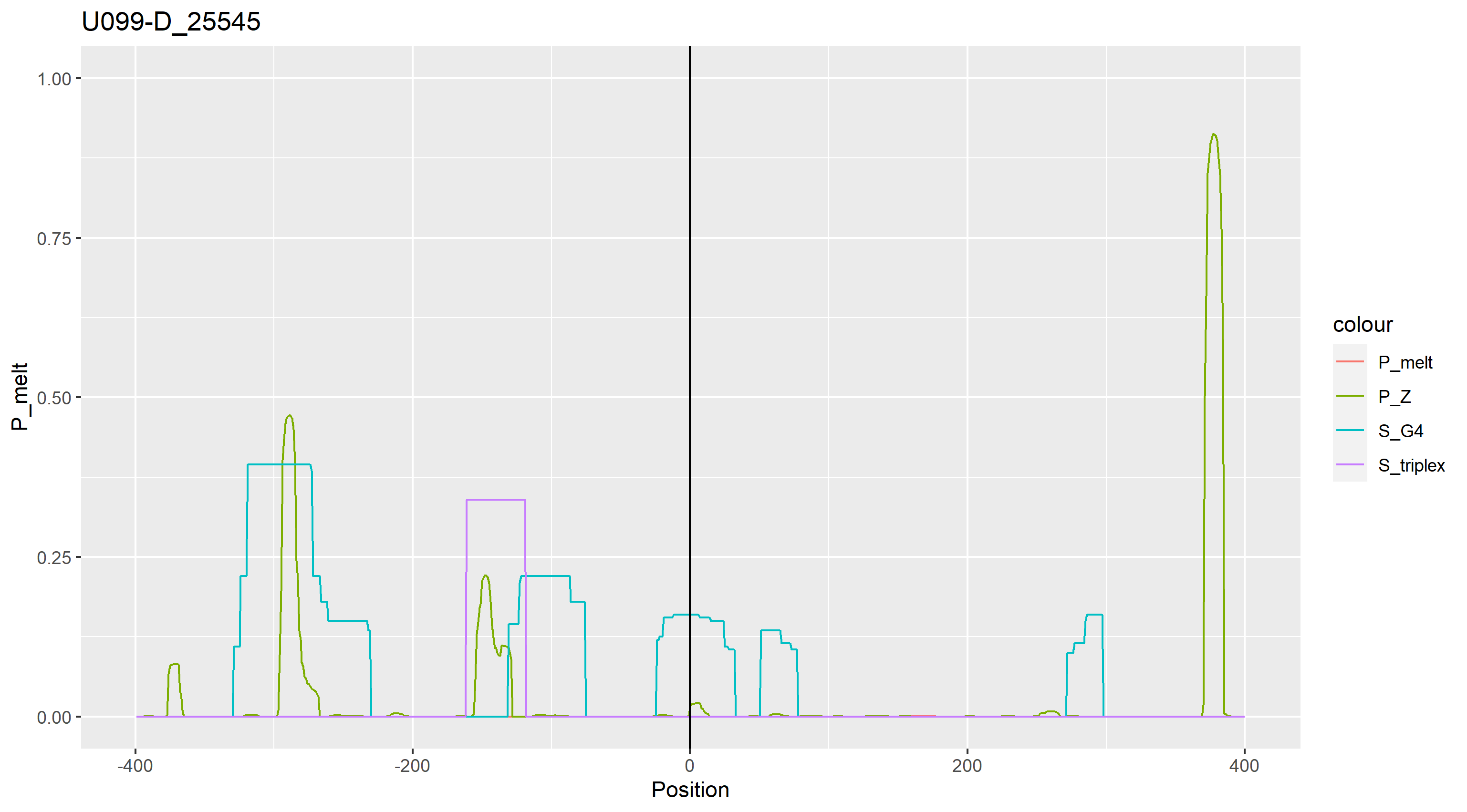

Supplement: S1 Graphs — The coordinate numbers in the figures of some breakpoints differ slightly from those in Column D of S2 Table because working draft genomes were used for non-B-DNA analyses, while S2 Table lists coordinates in the finished genomes uploaded to Genbank. The coordinate differences come from refinements in the genome termini and repeat regions, outside the analyzed sequences. (ZIP) [file ppat.1010524.s014.zip › Supplementary graphs/U099-D_25545.png]

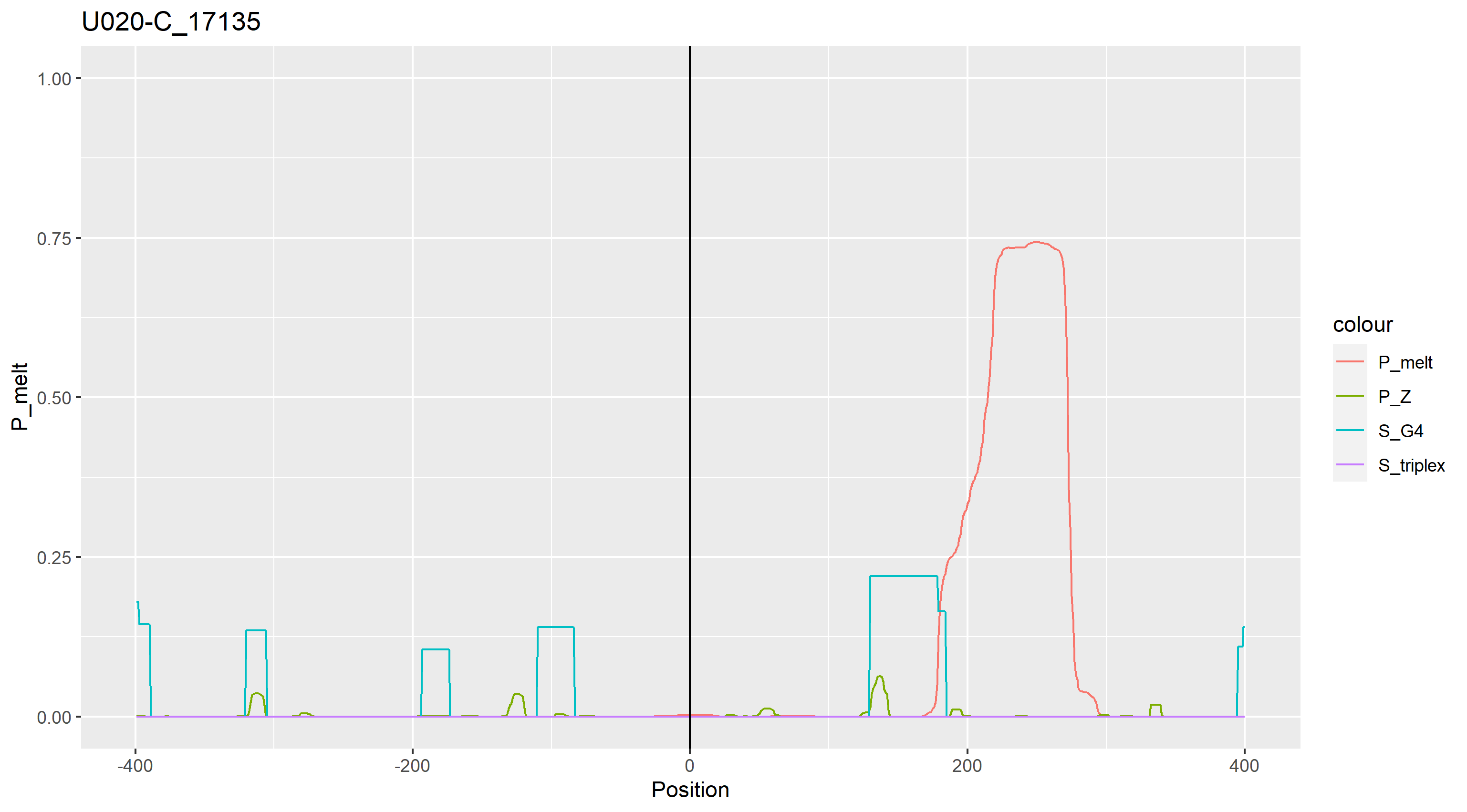

Supplement: S1 Graphs — The coordinate numbers in the figures of some breakpoints differ slightly from those in Column D of S2 Table because working draft genomes were used for non-B-DNA analyses, while S2 Table lists coordinates in the finished genomes uploaded to Genbank. The coordinate differences come from refinements in the genome termini and repeat regions, outside the analyzed sequences. (ZIP) [file ppat.1010524.s014.zip › Supplementary graphs/U020-C_17135.png]

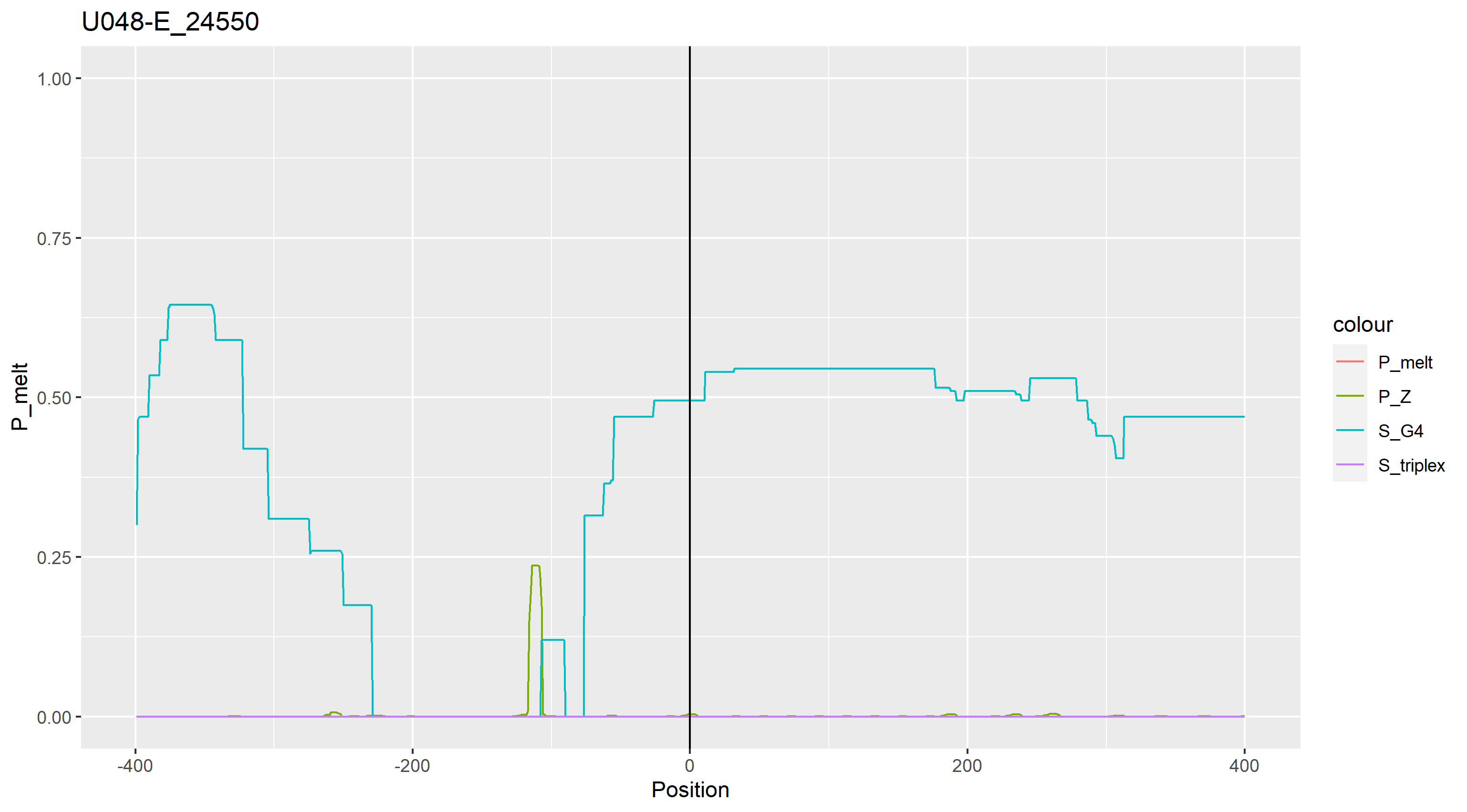

Supplement: S1 Graphs — The coordinate numbers in the figures of some breakpoints differ slightly from those in Column D of S2 Table because working draft genomes were used for non-B-DNA analyses, while S2 Table lists coordinates in the finished genomes uploaded to Genbank. The coordinate differences come from refinements in the genome termini and repeat regions, outside the analyzed sequences. (ZIP) [file ppat.1010524.s014.zip › Supplementary graphs/U048-E_24550.png]

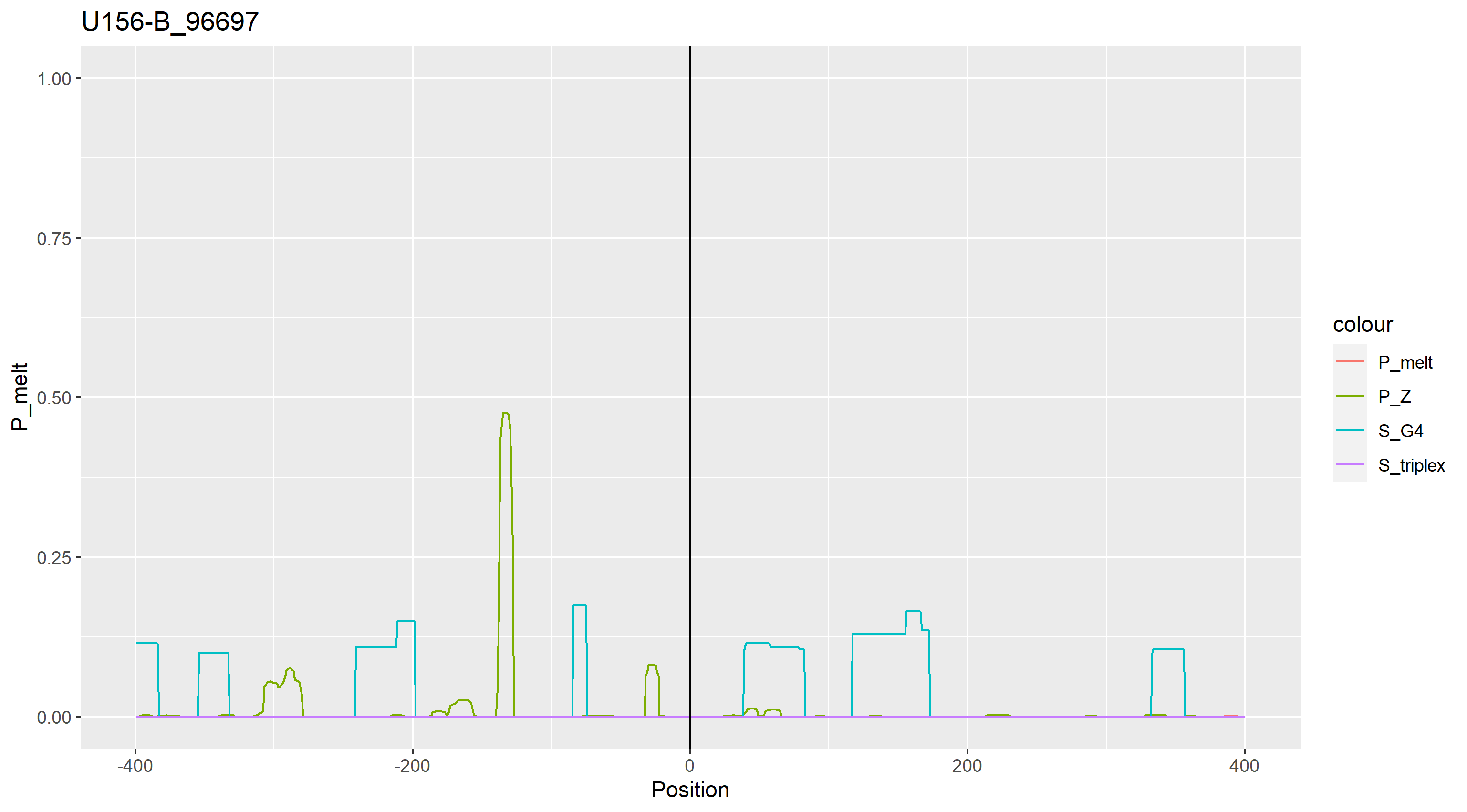

Supplement: S1 Graphs — The coordinate numbers in the figures of some breakpoints differ slightly from those in Column D of S2 Table because working draft genomes were used for non-B-DNA analyses, while S2 Table lists coordinates in the finished genomes uploaded to Genbank. The coordinate differences come from refinements in the genome termini and repeat regions, outside the analyzed sequences. (ZIP) [file ppat.1010524.s014.zip › Supplementary graphs/U156-B_96697.png]

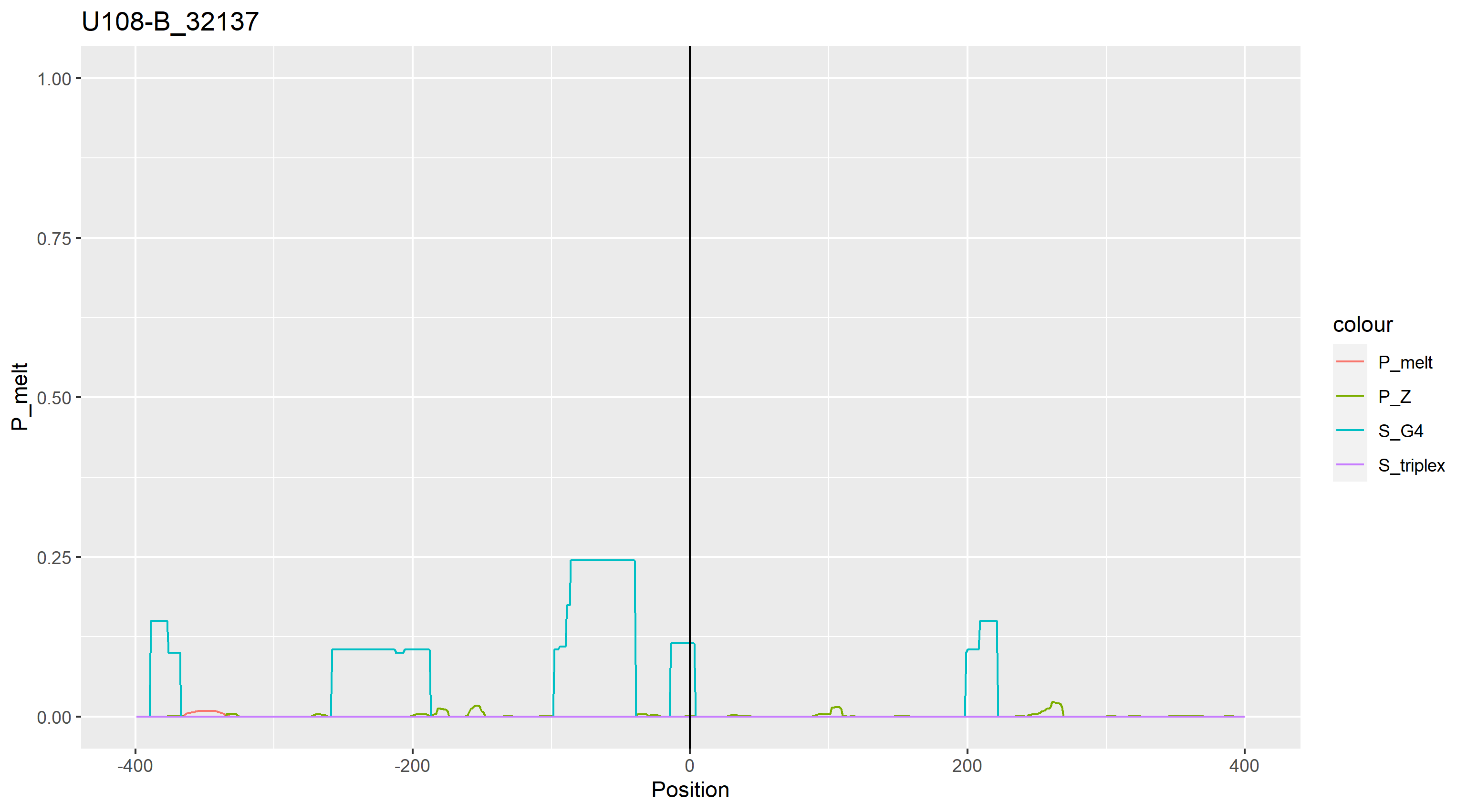

Supplement: S1 Graphs — The coordinate numbers in the figures of some breakpoints differ slightly from those in Column D of S2 Table because working draft genomes were used for non-B-DNA analyses, while S2 Table lists coordinates in the finished genomes uploaded to Genbank. The coordinate differences come from refinements in the genome termini and repeat regions, outside the analyzed sequences. (ZIP) [file ppat.1010524.s014.zip › Supplementary graphs/U108-B_32137.png]

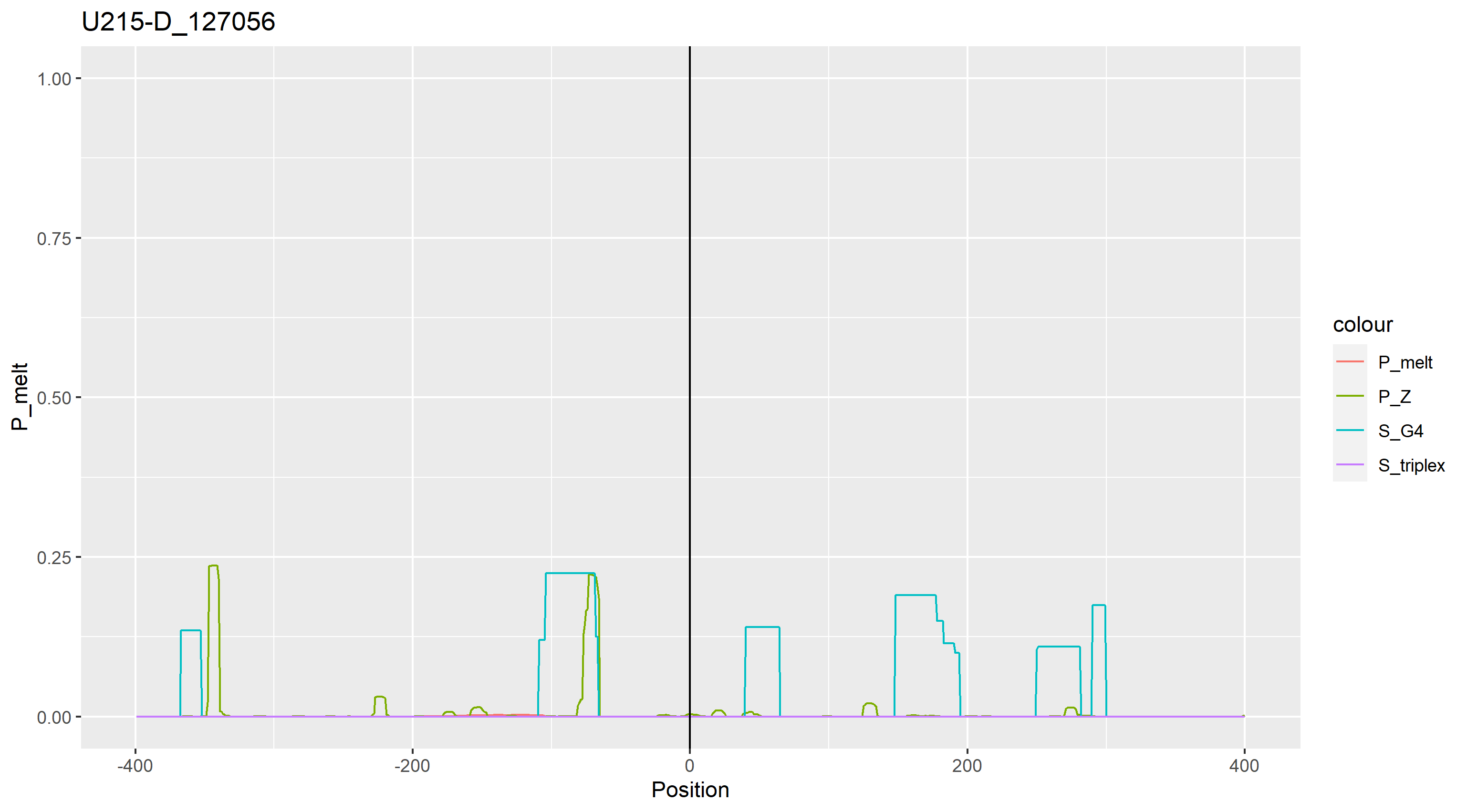

Supplement: S1 Graphs — The coordinate numbers in the figures of some breakpoints differ slightly from those in Column D of S2 Table because working draft genomes were used for non-B-DNA analyses, while S2 Table lists coordinates in the finished genomes uploaded to Genbank. The coordinate differences come from refinements in the genome termini and repeat regions, outside the analyzed sequences. (ZIP) [file ppat.1010524.s014.zip › Supplementary graphs/U215-D_127056.png]

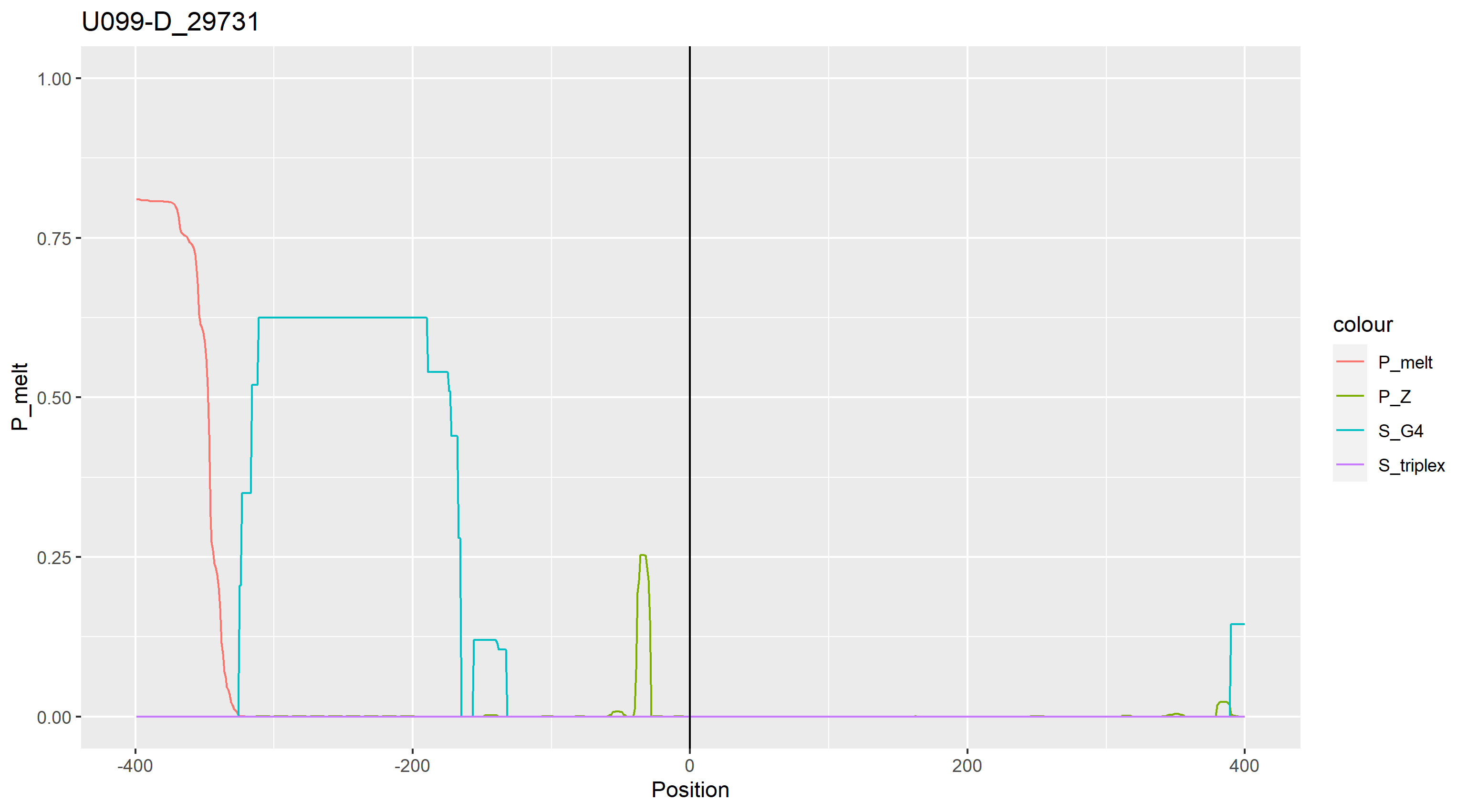

Supplement: S1 Graphs — The coordinate numbers in the figures of some breakpoints differ slightly from those in Column D of S2 Table because working draft genomes were used for non-B-DNA analyses, while S2 Table lists coordinates in the finished genomes uploaded to Genbank. The coordinate differences come from refinements in the genome termini and repeat regions, outside the analyzed sequences. (ZIP) [file ppat.1010524.s014.zip › Supplementary graphs/U099-D_29731.png]

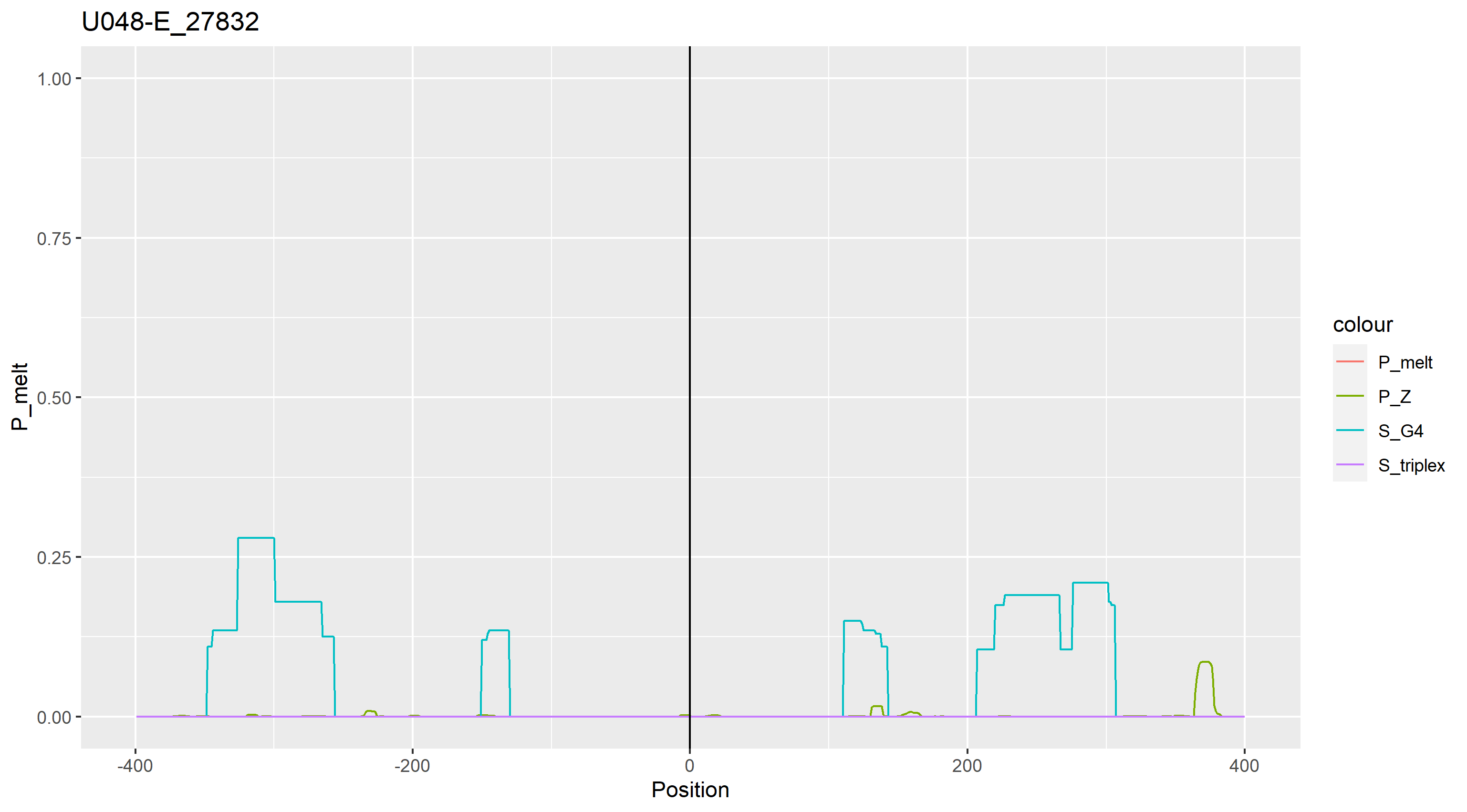

Supplement: S1 Graphs — The coordinate numbers in the figures of some breakpoints differ slightly from those in Column D of S2 Table because working draft genomes were used for non-B-DNA analyses, while S2 Table lists coordinates in the finished genomes uploaded to Genbank. The coordinate differences come from refinements in the genome termini and repeat regions, outside the analyzed sequences. (ZIP) [file ppat.1010524.s014.zip › Supplementary graphs/U048-E_27832.png]

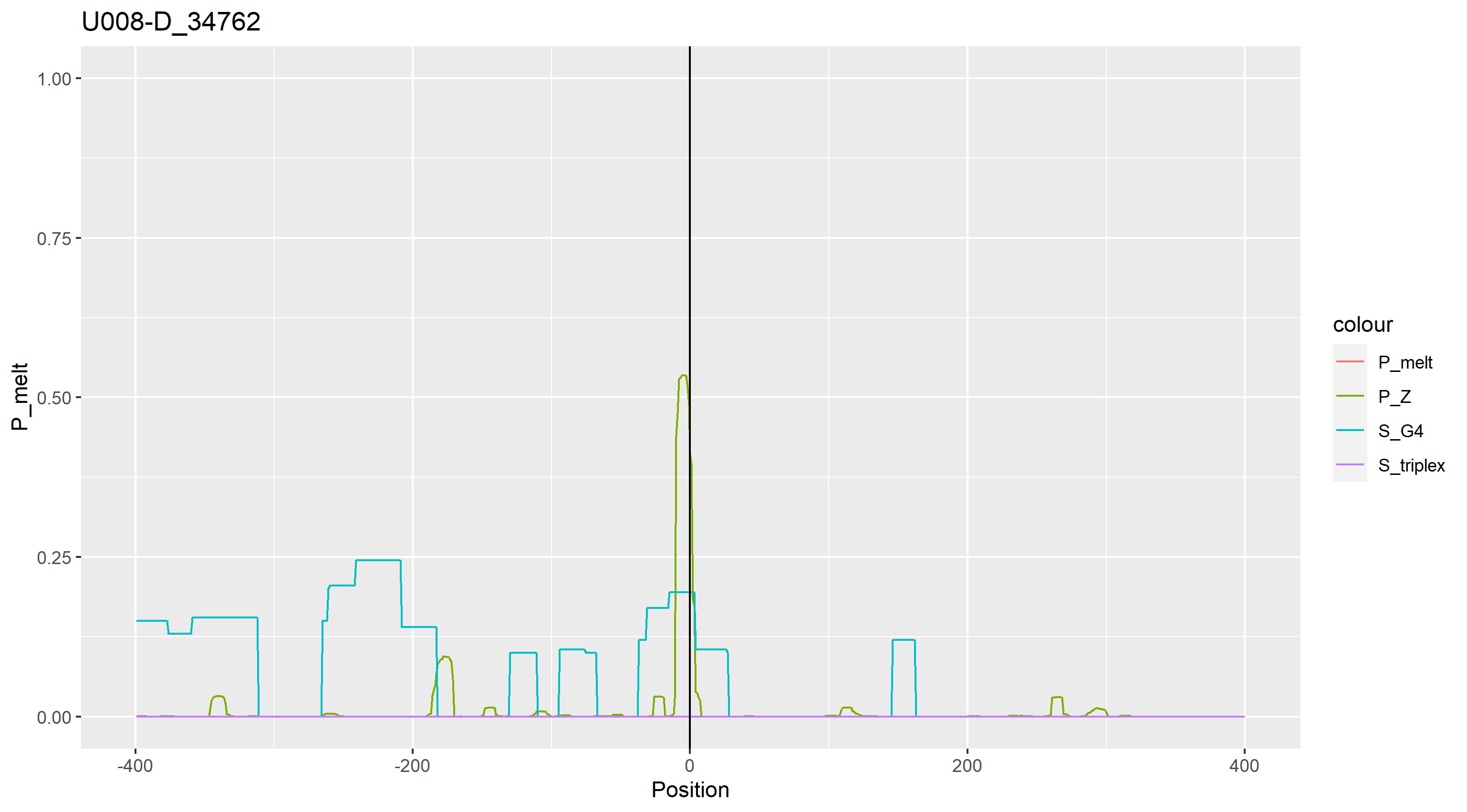

Supplement: S1 Graphs — The coordinate numbers in the figures of some breakpoints differ slightly from those in Column D of S2 Table because working draft genomes were used for non-B-DNA analyses, while S2 Table lists coordinates in the finished genomes uploaded to Genbank. The coordinate differences come from refinements in the genome termini and repeat regions, outside the analyzed sequences. (ZIP) [file ppat.1010524.s014.zip › Supplementary graphs/U008-D_34762.png]

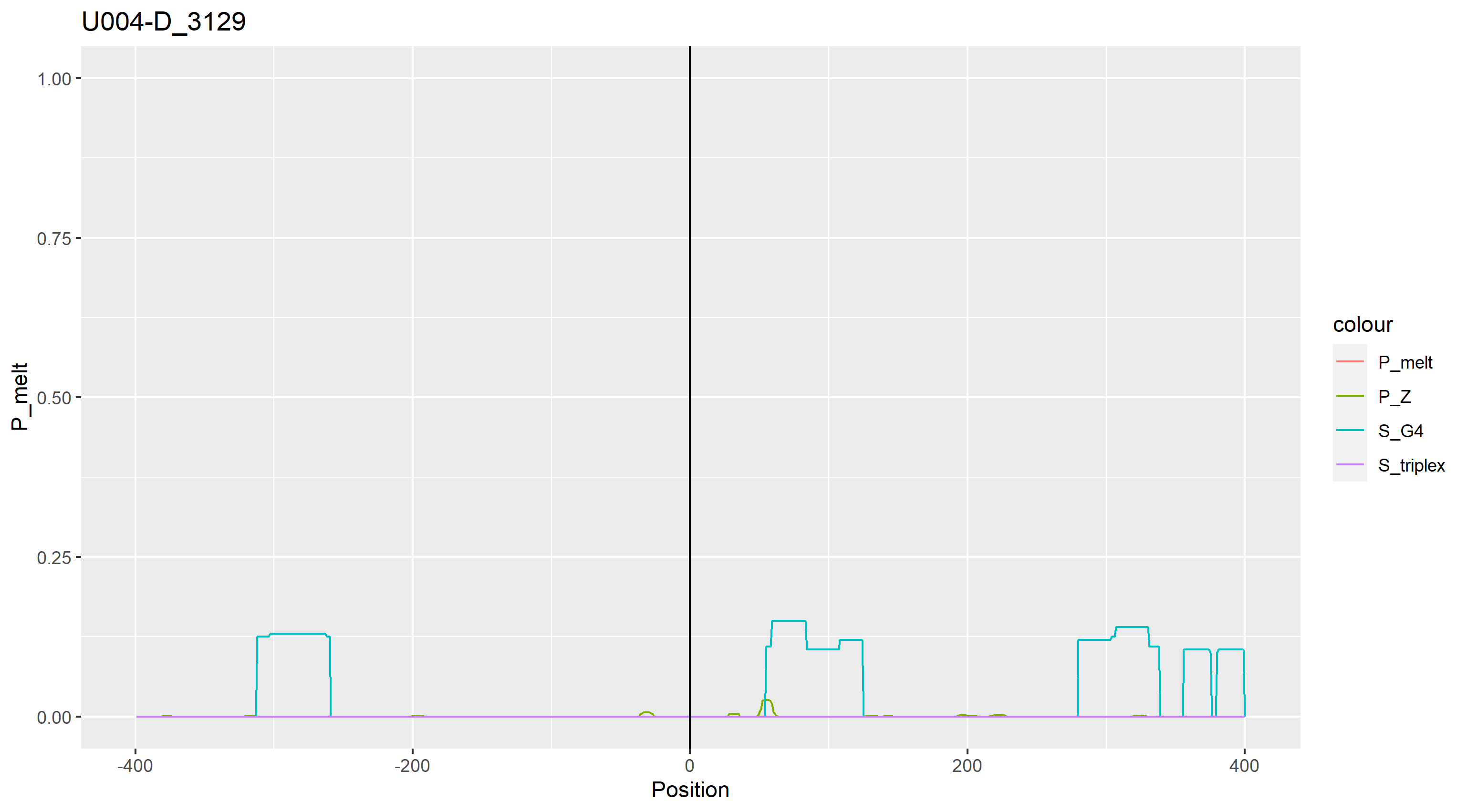

Supplement: S1 Graphs — The coordinate numbers in the figures of some breakpoints differ slightly from those in Column D of S2 Table because working draft genomes were used for non-B-DNA analyses, while S2 Table lists coordinates in the finished genomes uploaded to Genbank. The coordinate differences come from refinements in the genome termini and repeat regions, outside the analyzed sequences. (ZIP) [file ppat.1010524.s014.zip › Supplementary graphs/U004-D_3129.png]

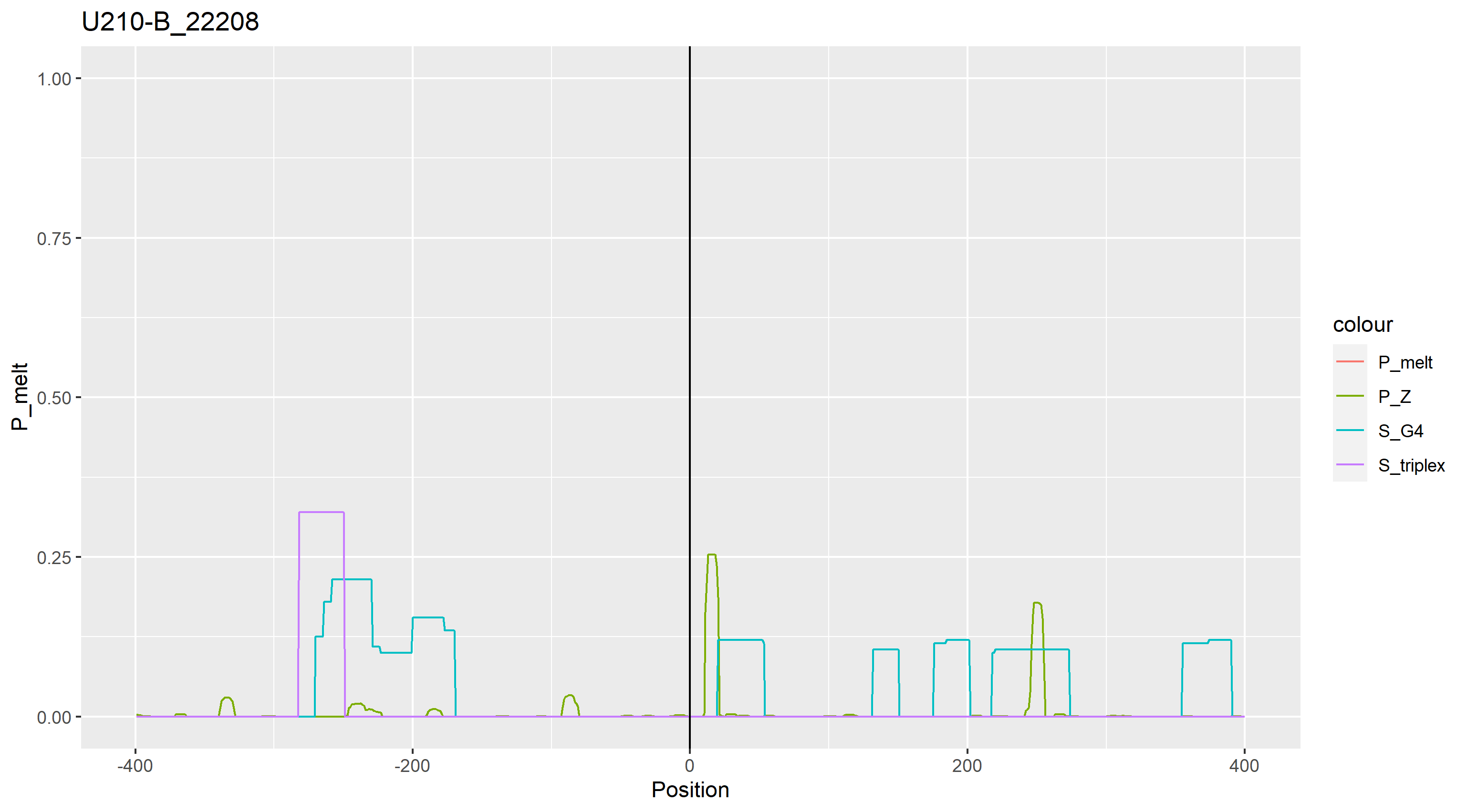

Supplement: S1 Graphs — The coordinate numbers in the figures of some breakpoints differ slightly from those in Column D of S2 Table because working draft genomes were used for non-B-DNA analyses, while S2 Table lists coordinates in the finished genomes uploaded to Genbank. The coordinate differences come from refinements in the genome termini and repeat regions, outside the analyzed sequences. (ZIP) [file ppat.1010524.s014.zip › Supplementary graphs/U210-B_22208.png]

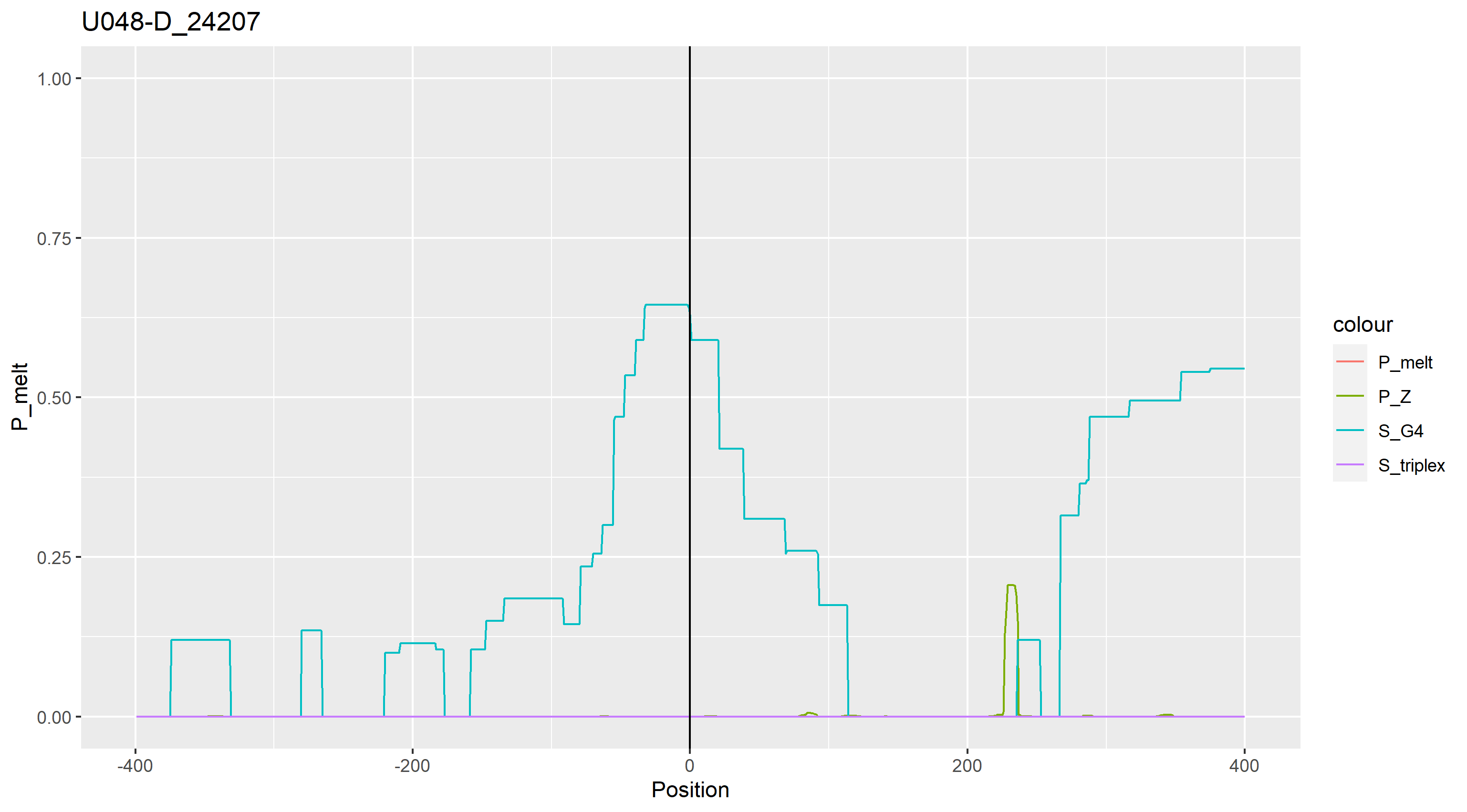

Supplement: S1 Graphs — The coordinate numbers in the figures of some breakpoints differ slightly from those in Column D of S2 Table because working draft genomes were used for non-B-DNA analyses, while S2 Table lists coordinates in the finished genomes uploaded to Genbank. The coordinate differences come from refinements in the genome termini and repeat regions, outside the analyzed sequences. (ZIP) [file ppat.1010524.s014.zip › Supplementary graphs/U048-D_24207.png]

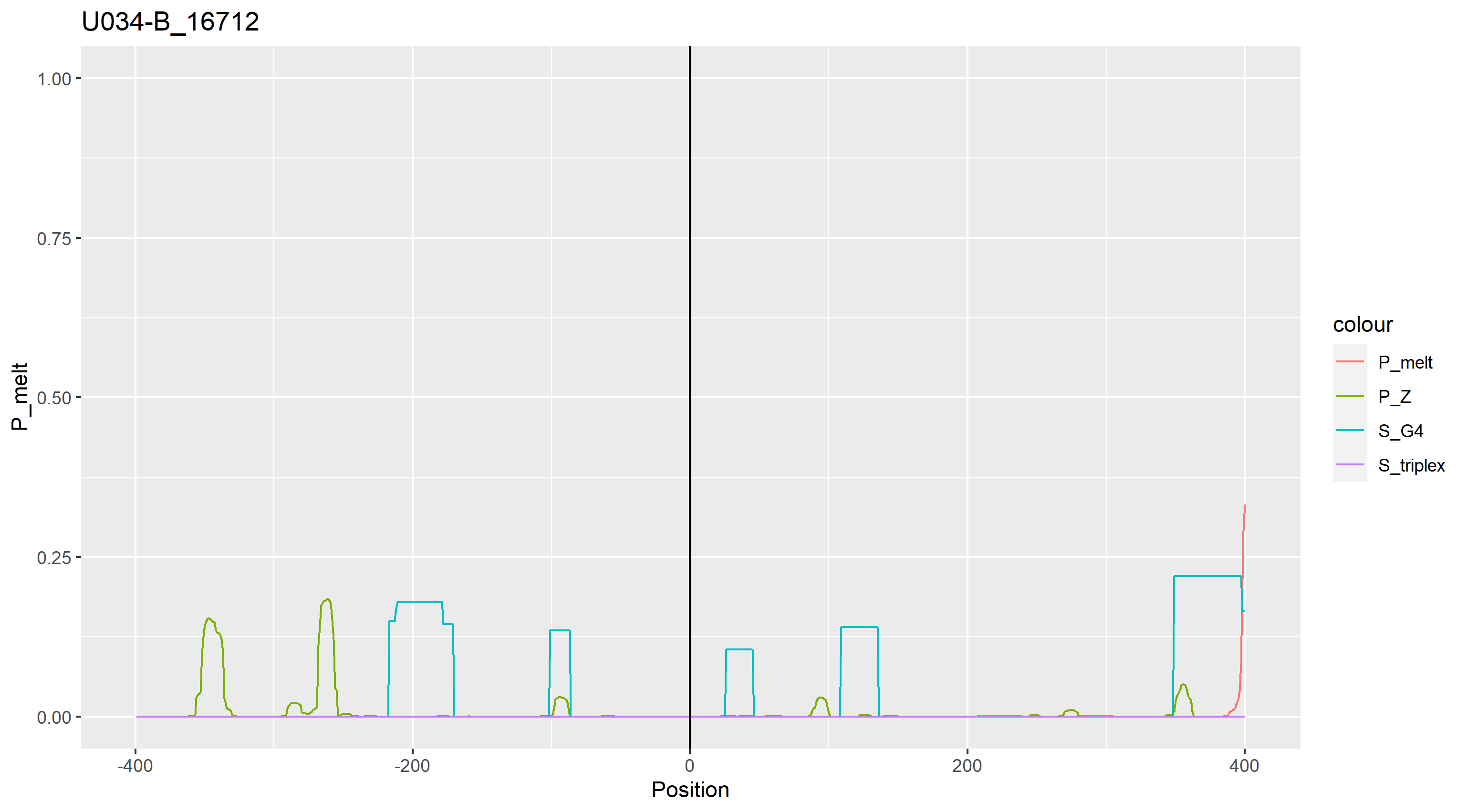

Supplement: S1 Graphs — The coordinate numbers in the figures of some breakpoints differ slightly from those in Column D of S2 Table because working draft genomes were used for non-B-DNA analyses, while S2 Table lists coordinates in the finished genomes uploaded to Genbank. The coordinate differences come from refinements in the genome termini and repeat regions, outside the analyzed sequences. (ZIP) [file ppat.1010524.s014.zip › Supplementary graphs/U034-B_16712.png]

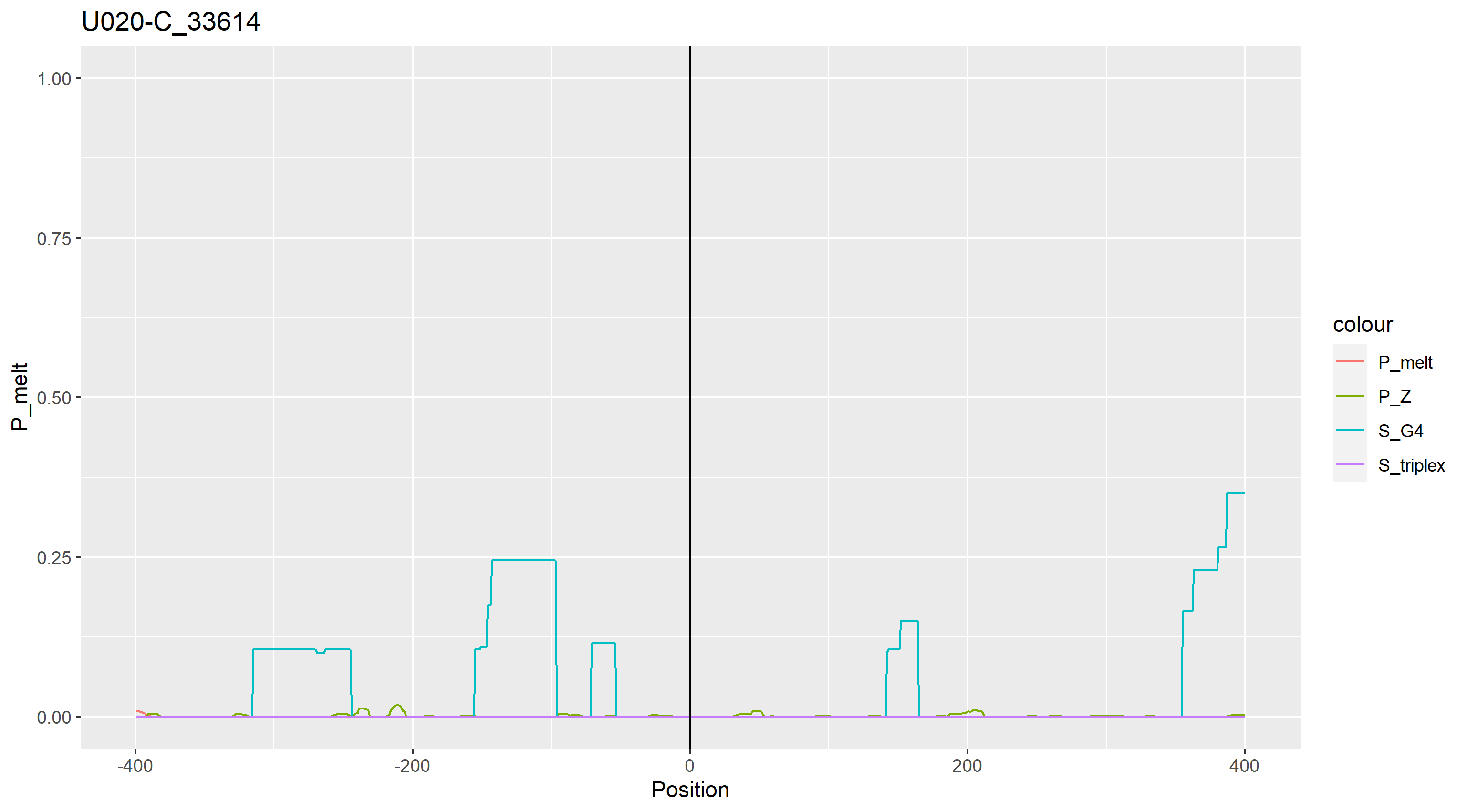

Supplement: S1 Graphs — The coordinate numbers in the figures of some breakpoints differ slightly from those in Column D of S2 Table because working draft genomes were used for non-B-DNA analyses, while S2 Table lists coordinates in the finished genomes uploaded to Genbank. The coordinate differences come from refinements in the genome termini and repeat regions, outside the analyzed sequences. (ZIP) [file ppat.1010524.s014.zip › Supplementary graphs/U020-C_33614.png]

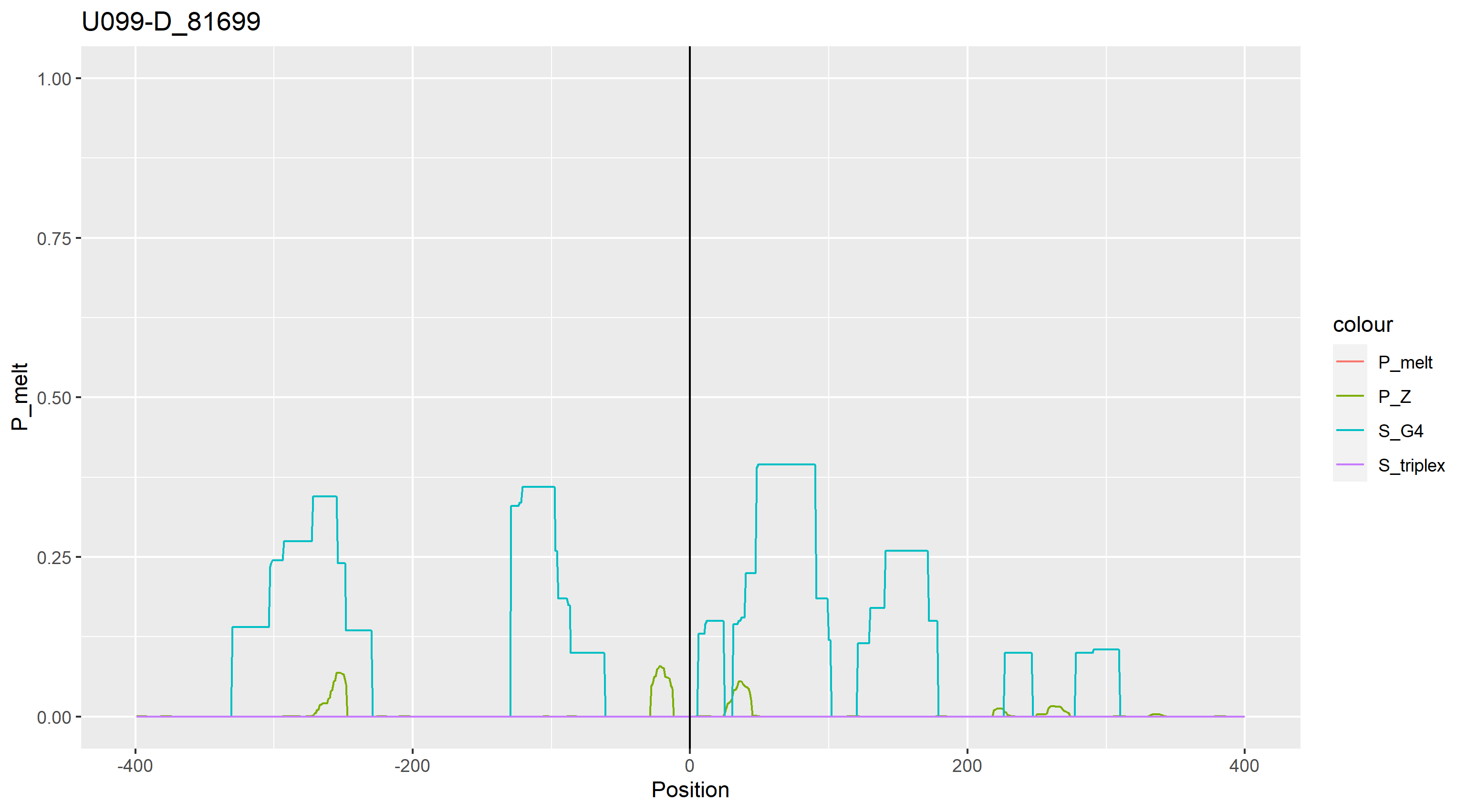

Supplement: S1 Graphs — The coordinate numbers in the figures of some breakpoints differ slightly from those in Column D of S2 Table because working draft genomes were used for non-B-DNA analyses, while S2 Table lists coordinates in the finished genomes uploaded to Genbank. The coordinate differences come from refinements in the genome termini and repeat regions, outside the analyzed sequences. (ZIP) [file ppat.1010524.s014.zip › Supplementary graphs/U099-D_81699.png]

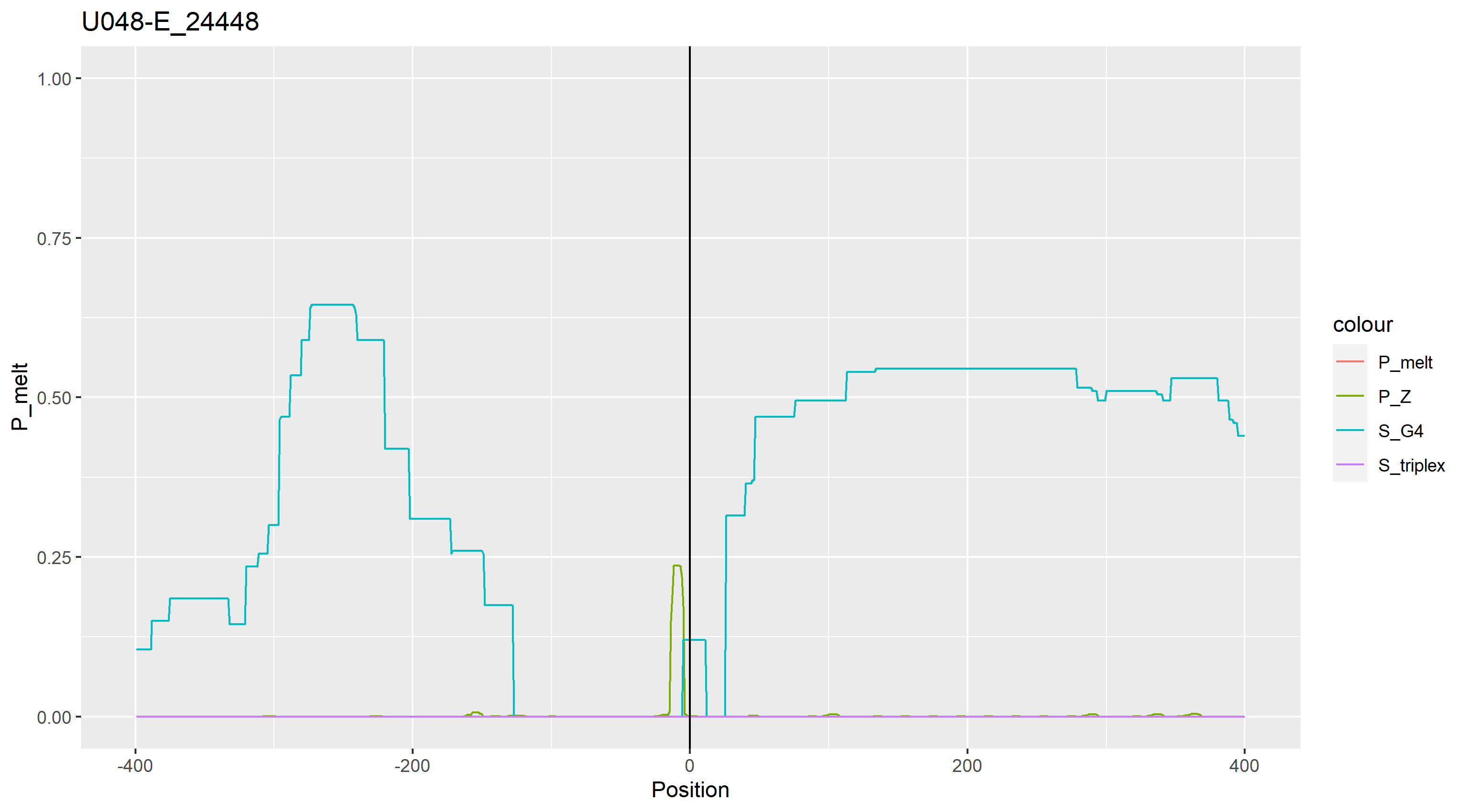

Supplement: S1 Graphs — The coordinate numbers in the figures of some breakpoints differ slightly from those in Column D of S2 Table because working draft genomes were used for non-B-DNA analyses, while S2 Table lists coordinates in the finished genomes uploaded to Genbank. The coordinate differences come from refinements in the genome termini and repeat regions, outside the analyzed sequences. (ZIP) [file ppat.1010524.s014.zip › Supplementary graphs/U048-E_24448.png]

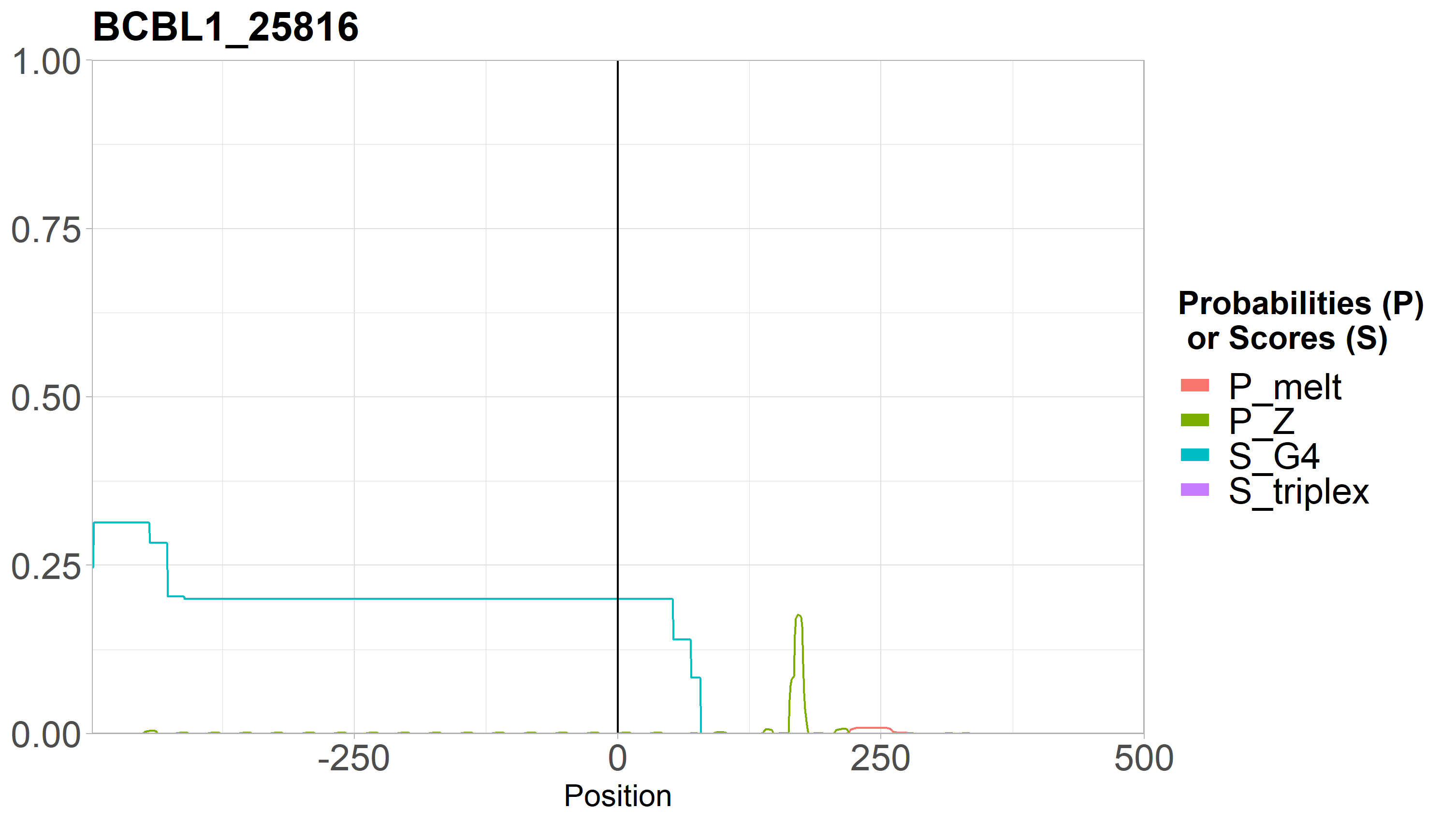

Supplement: S1 Graphs — The coordinate numbers in the figures of some breakpoints differ slightly from those in Column D of S2 Table because working draft genomes were used for non-B-DNA analyses, while S2 Table lists coordinates in the finished genomes uploaded to Genbank. The coordinate differences come from refinements in the genome termini and repeat regions, outside the analyzed sequences. (ZIP) [file ppat.1010524.s014.zip › Supplementary graphs/BCBL1_25816.png]

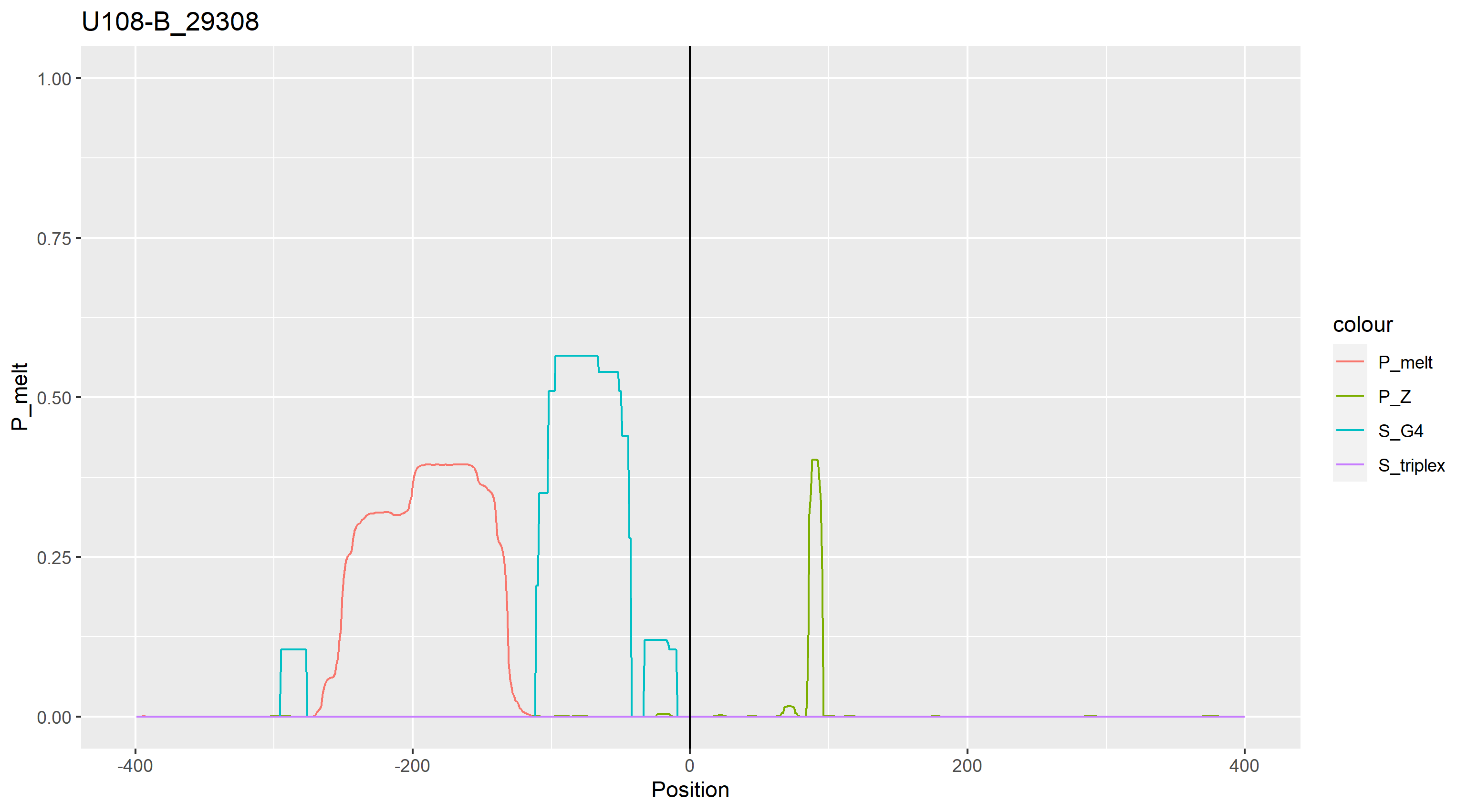

Supplement: S1 Graphs — The coordinate numbers in the figures of some breakpoints differ slightly from those in Column D of S2 Table because working draft genomes were used for non-B-DNA analyses, while S2 Table lists coordinates in the finished genomes uploaded to Genbank. The coordinate differences come from refinements in the genome termini and repeat regions, outside the analyzed sequences. (ZIP) [file ppat.1010524.s014.zip › Supplementary graphs/U108-B_29308.png]

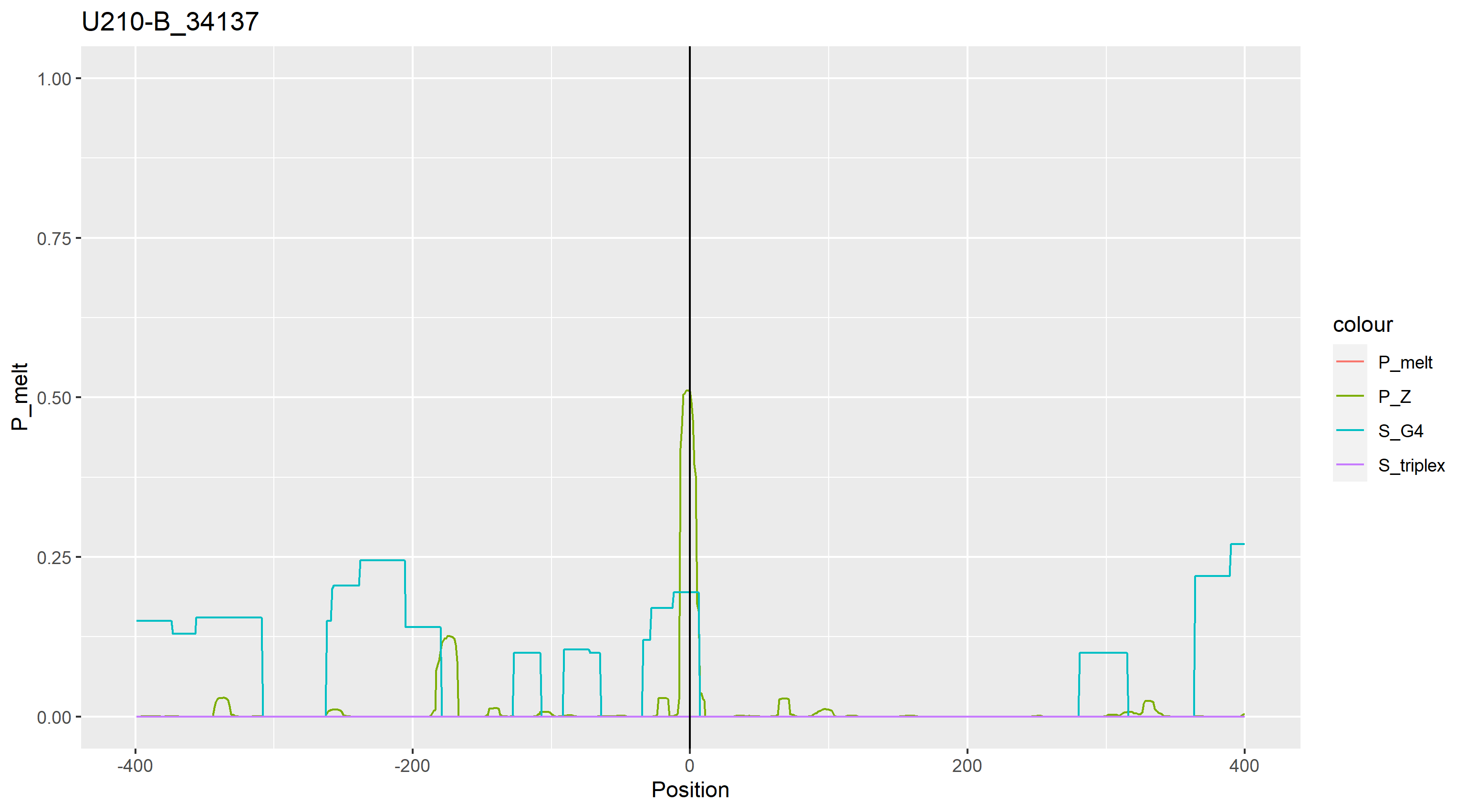

Supplement: S1 Graphs — The coordinate numbers in the figures of some breakpoints differ slightly from those in Column D of S2 Table because working draft genomes were used for non-B-DNA analyses, while S2 Table lists coordinates in the finished genomes uploaded to Genbank. The coordinate differences come from refinements in the genome termini and repeat regions, outside the analyzed sequences. (ZIP) [file ppat.1010524.s014.zip › Supplementary graphs/U210-B_34137.png]

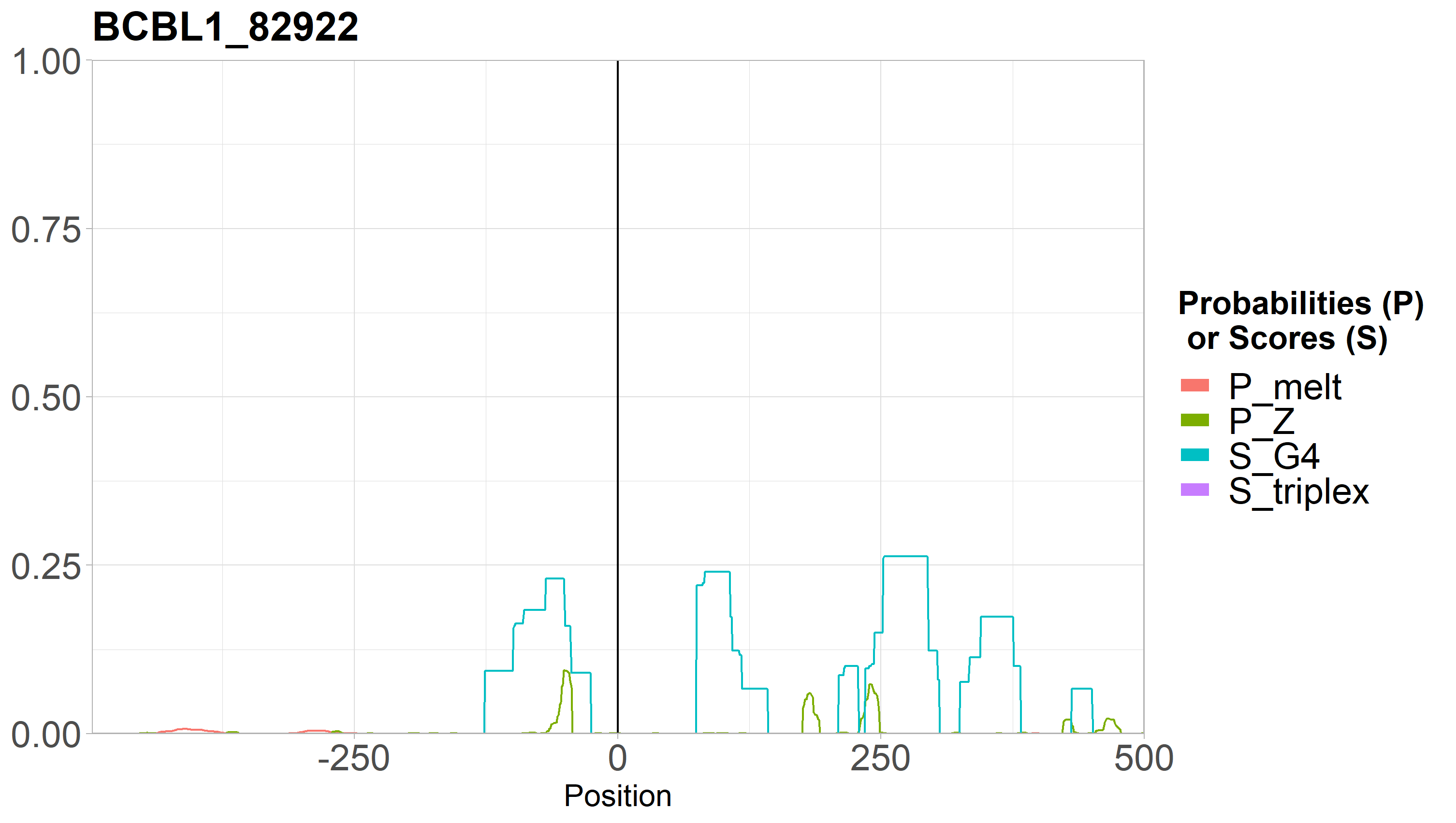

Supplement: S1 Graphs — The coordinate numbers in the figures of some breakpoints differ slightly from those in Column D of S2 Table because working draft genomes were used for non-B-DNA analyses, while S2 Table lists coordinates in the finished genomes uploaded to Genbank. The coordinate differences come from refinements in the genome termini and repeat regions, outside the analyzed sequences. (ZIP) [file ppat.1010524.s014.zip › Supplementary graphs/BCBL1_82922.png]

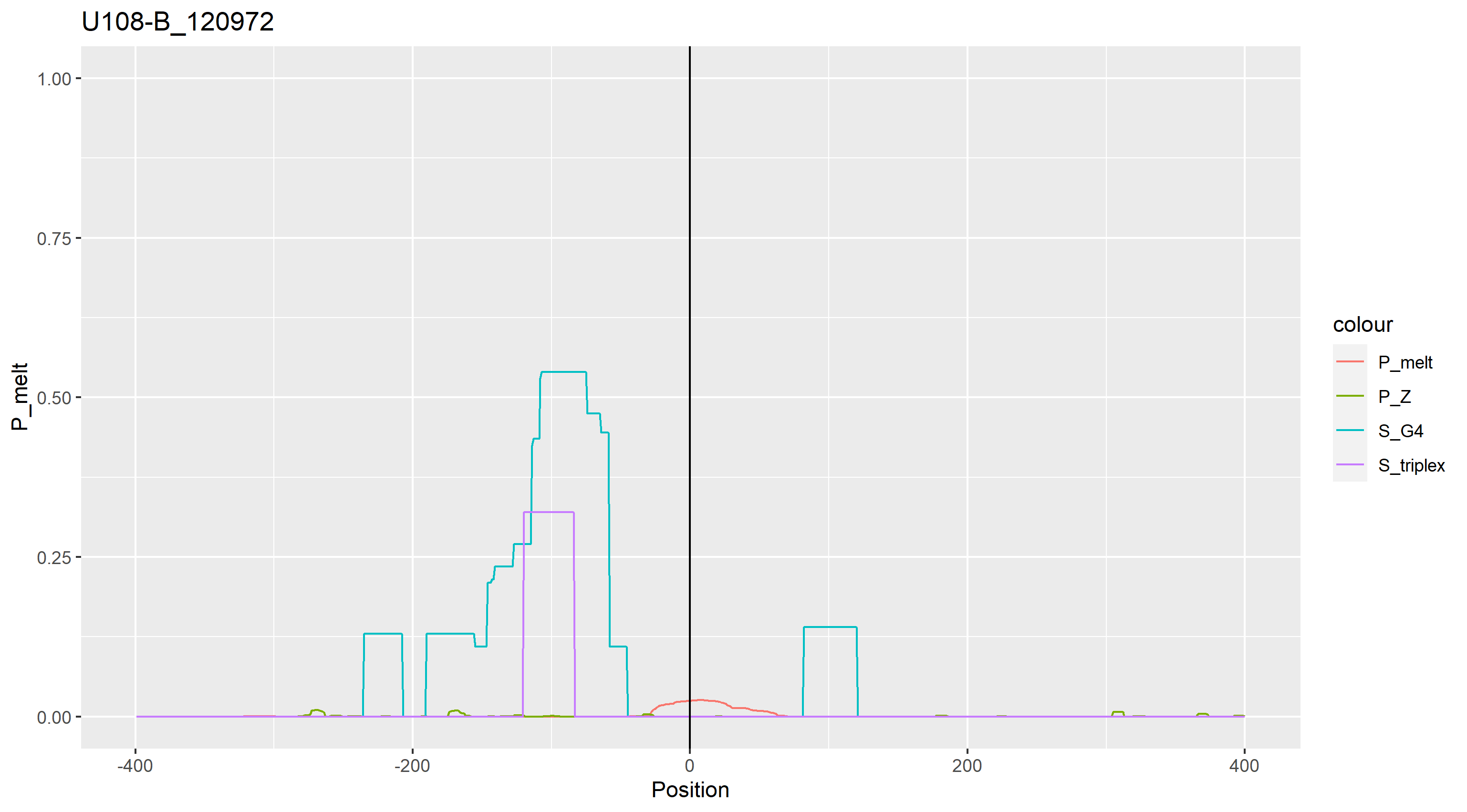

Supplement: S1 Graphs — The coordinate numbers in the figures of some breakpoints differ slightly from those in Column D of S2 Table because working draft genomes were used for non-B-DNA analyses, while S2 Table lists coordinates in the finished genomes uploaded to Genbank. The coordinate differences come from refinements in the genome termini and repeat regions, outside the analyzed sequences. (ZIP) [file ppat.1010524.s014.zip › Supplementary graphs/U108-B_120972.png]

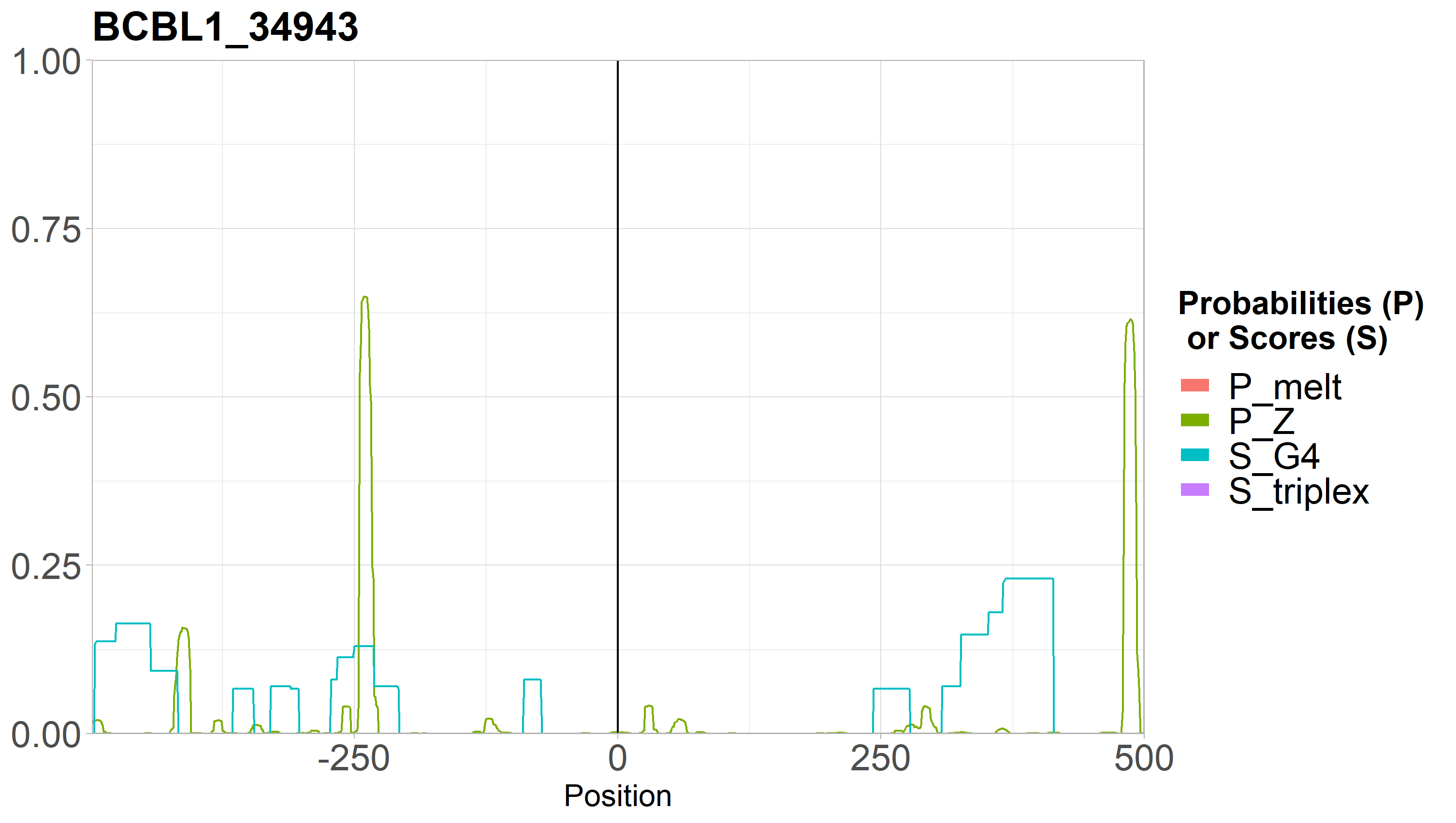

Supplement: S1 Graphs — The coordinate numbers in the figures of some breakpoints differ slightly from those in Column D of S2 Table because working draft genomes were used for non-B-DNA analyses, while S2 Table lists coordinates in the finished genomes uploaded to Genbank. The coordinate differences come from refinements in the genome termini and repeat regions, outside the analyzed sequences. (ZIP) [file ppat.1010524.s014.zip › Supplementary graphs/BCBL1_34943.png]

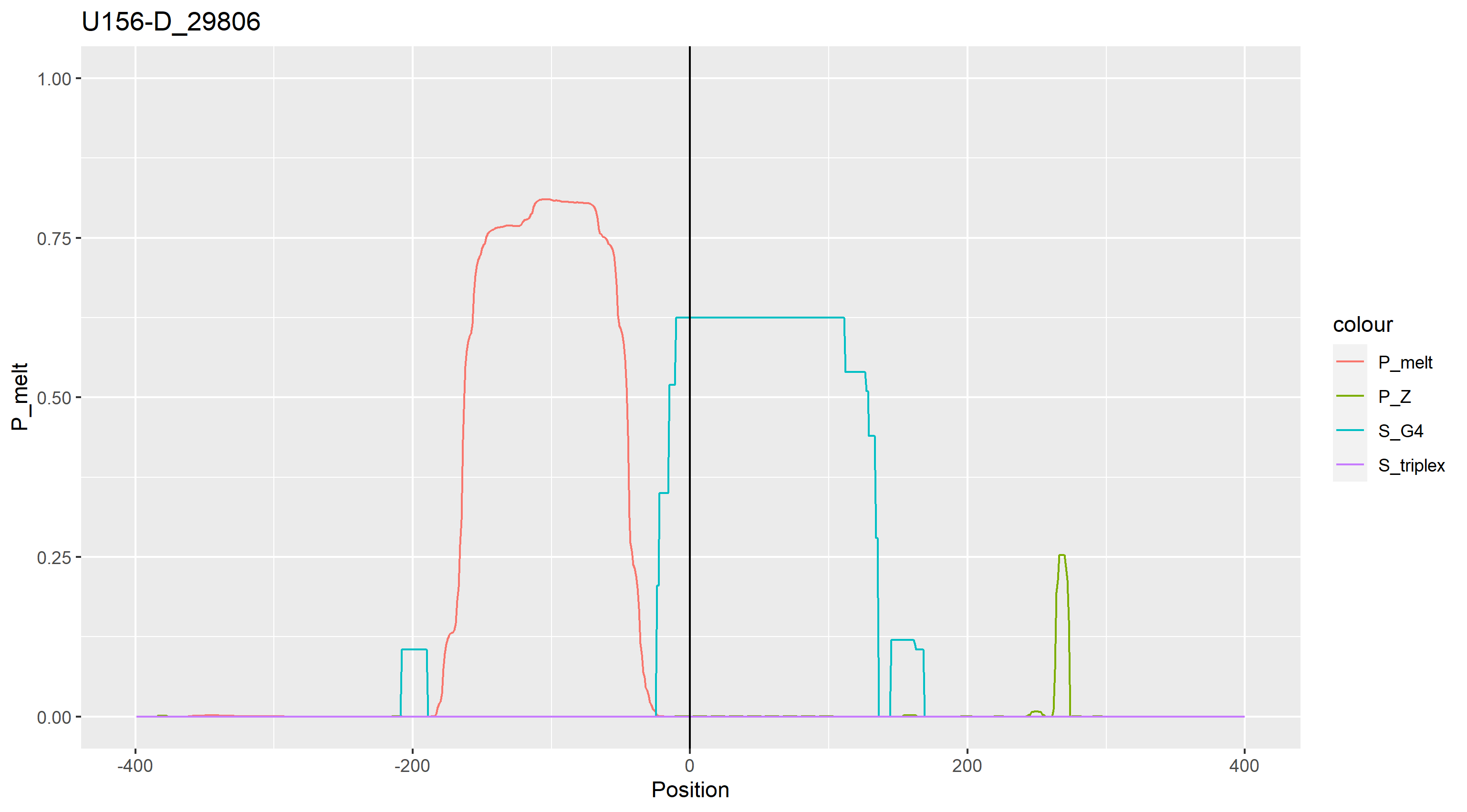

Supplement: S1 Graphs — The coordinate numbers in the figures of some breakpoints differ slightly from those in Column D of S2 Table because working draft genomes were used for non-B-DNA analyses, while S2 Table lists coordinates in the finished genomes uploaded to Genbank. The coordinate differences come from refinements in the genome termini and repeat regions, outside the analyzed sequences. (ZIP) [file ppat.1010524.s014.zip › Supplementary graphs/U156-D_29806.png]

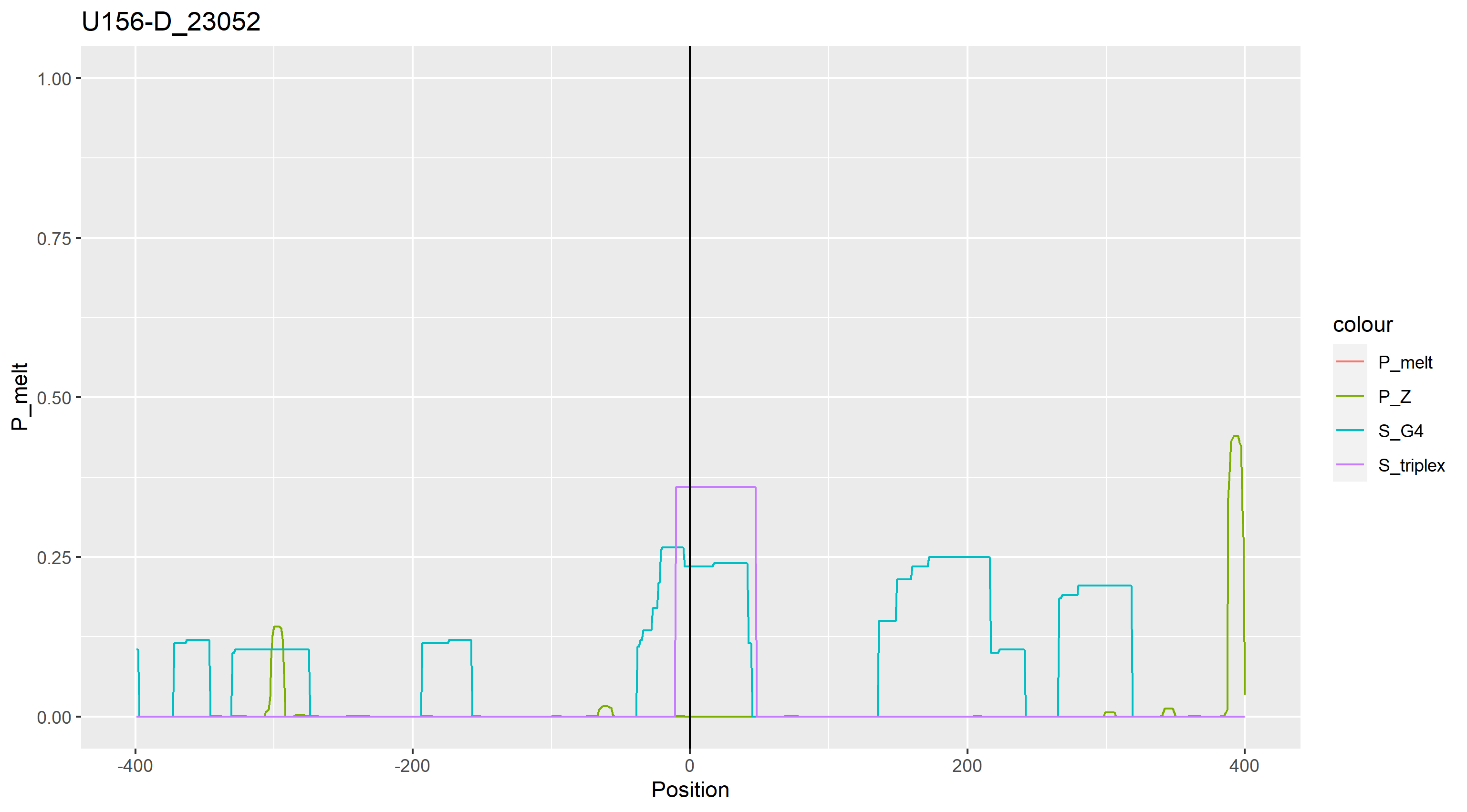

Supplement: S1 Graphs — The coordinate numbers in the figures of some breakpoints differ slightly from those in Column D of S2 Table because working draft genomes were used for non-B-DNA analyses, while S2 Table lists coordinates in the finished genomes uploaded to Genbank. The coordinate differences come from refinements in the genome termini and repeat regions, outside the analyzed sequences. (ZIP) [file ppat.1010524.s014.zip › Supplementary graphs/U156-D_23052.png]

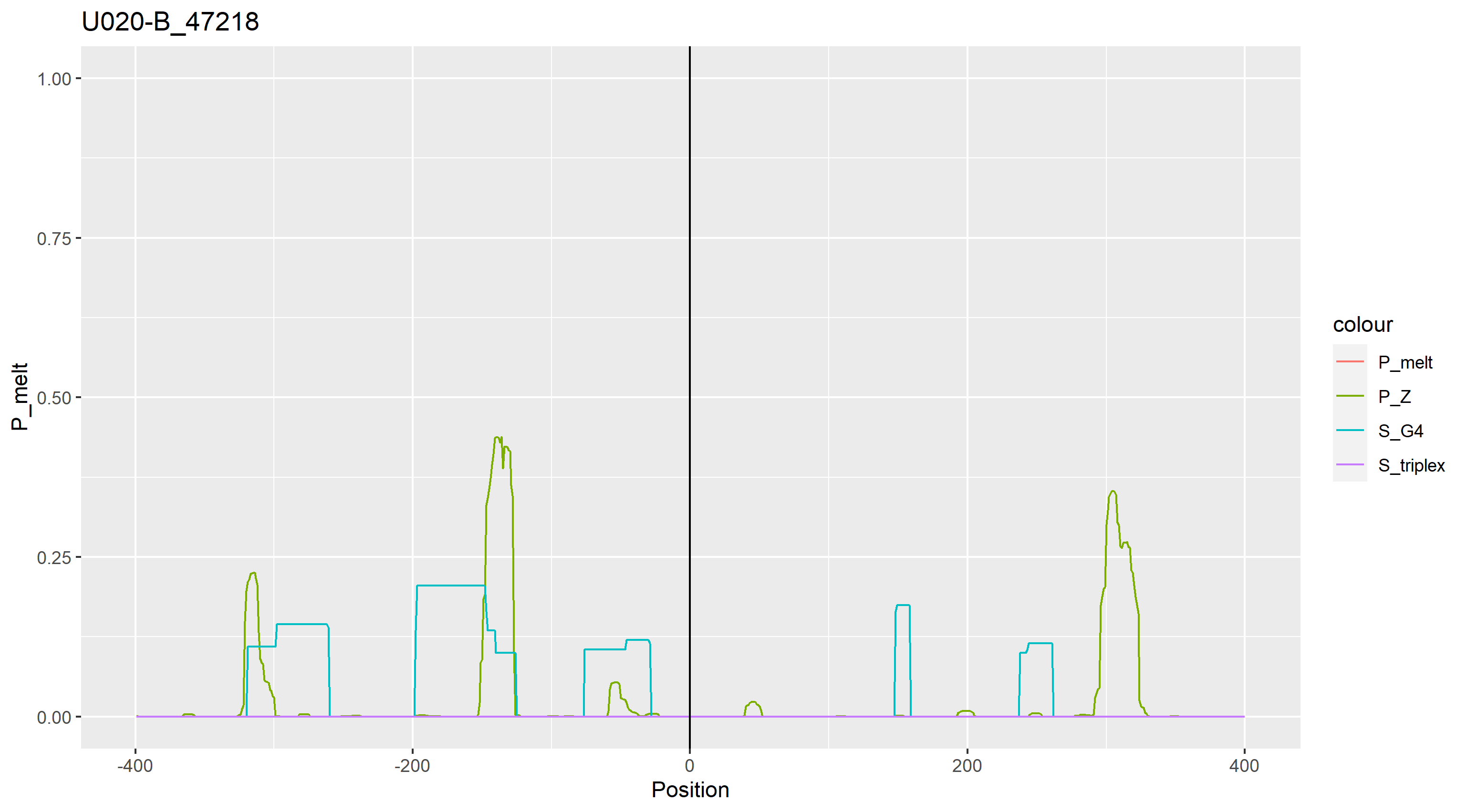

Supplement: S1 Graphs — The coordinate numbers in the figures of some breakpoints differ slightly from those in Column D of S2 Table because working draft genomes were used for non-B-DNA analyses, while S2 Table lists coordinates in the finished genomes uploaded to Genbank. The coordinate differences come from refinements in the genome termini and repeat regions, outside the analyzed sequences. (ZIP) [file ppat.1010524.s014.zip › Supplementary graphs/U020-B_47218.png]
