# Supplementary material for: Genomic changes in Kaposi Sarcoma-associated Herpesvirus and their clinical correlates
Source: PLoS Pathog. 2022 Nov 28;18(11):e1010524. doi: 10.1371/journal.ppat.1010524 (PMC9731496; doi:10.1371/journal.ppat.1010524)
Supplement: Attachment — Submitted filename: ResponseToReviewer comments - KSHV WGS_8-19.docx [file ppat.1010524.s015.docx]

***Responses to Reviewer's Questions are in bold italics. Noted changes in the manuscript are highlighted.***

**Part I - Summary**

**Reviewer #1:** This is an interesting paper by Santiago and others to study the KSHV genome variations using NGS of the whole genome of the Kaposi’s sarcoma herpesvirus (KSHV) of tumor DNA obtained from a cohort of patients from Uganda. Sixty-five tumors from 29 individuals were analyzed in this study. The authors found that there were frequent over coverage by ddPCR and sequence reads that the region covering K5 and K6 have higher coverage than the rest of the KSHV genome from the same tumors, suggesting multiple copies of the genes within the genome, supporting their previous findings. In addition, there were alternations, rearrangements and mutations in the K8.1 and the miR-K10 microRNA coding sequences.

This is a very detailed and extensive study as a follow up of a study with a smaller number of tumors by the same group and several studies from other groups. The results presented here supported most of their earlier findings. This study is interesting and potentially important because KSHV genome variations and de novo mutation may contribute the biology of the virus and disease pathogenesis. However, there are a number of concerns with the study and its analysis. In addition, there are some discrepancies throughout.

1) A major concern with the study is over representations, differential copy numbers and variations in a number of KSHV genes from the same tumor, as indicated by both ddPCR and genome sequencing. As shown in Table 2, there are substantial differences in the copy numbers between K2, ORF16, ORF50 or ORF73. For sample U020-E, there was only 1 copy of ORF50, but 172 copies of ORF73. It will be difficult to imagine that they could exist within the same genome. There is a need to demonstrate that such duplication occurs in the same genome in order to support the biological significance of gene duplication in the biology of the virus and tumorigenesis.

***Because of the short reads afforded by Illumina sequencing, we are unable to demonstrate that each of the duplications or deletions occurred in the same genome, although we were able to demonstrate breakpoints in many instances. In U020-E for example, no whole genome sequencing was conducted and thus the potentially complete loss of the ORF50 amplicon suggests the presence of very low levels or no potentially intact genomes within the same tumor biopsy (please note that the Table key indicates that a value of 1 indicates that the result was below the limit of detection). Heterogenous populations of potentially intact as well as aberrant viral genomes within a single biopsy was indeed shown in multiple cases. However, we do not think that the presence of aberrations being confined to singular genomic structures is needed to infer biological significance or for hypothesis generation. We have modified the text to add the sentence “*Viral genomic structures within tumor biopsies were heterogeneous, although in multiple instances, genomes with aberrant structures were more abundant than apparently intact viral genomes” *to the* Discussion *(Lines 485 – 487).***

2) The mutations in the miR-K10, and deletions and rearrangements of K8.1 genes are consistent and support other previous studies, it is possible that they could be an outcome of tumor cell and KSHV genome replication, rather than they playing a causative role in KS development.

***Yes, this is possible. We have tried to be careful in our wording and raised KSHV replication in tumor cells and causation among the possibilities. To clarify further, we modified and added to the final paragraph of the Discussion* *(Lines 627-631).***

3) The informatics workflow of the study was very informative, yet they could be more detailed and supported, based on the numerous tools that were used, and the fact that the KSHV genomes were de novo assembled.

***We referred to our earlier paper for the bioinformatics pipeline and tools used to generate the KSHV genomes* de novo *and also detailed any new methods. We are not sure what else would be useful without being repetitive.***

4) Their claims were not always supported by their figures. For instance, where they claim "G4 scores also peaked at the breakpoint position but not in the control data", however, that does not seem to be the case in the figure for regions in Fig 4D (roughly) around 225-250 nt.

***In figure 4D, the breakpoint is position 0, as indicated by the vertical line. This is where the largest peak was observed in the tumors, whereas there was no signal in the control data at this position. The reviewer refers to a peak 225-250 nt away from the breakpoint. It is to be expected that some regions in the control random dataset would have higher scores than in the observed set.***

5) What is the rationale behind their associations between non-B DNA structures (through their predicted sequence features) and rearrangement breakpoints? It could be better to calculate an overall predicted Z-DNA, such that, instead of permuting the breakpoint sequence interval, cruciform, melt motifs, etc., they could pick random regions of the same length that are not within rearrangement breakpoints, and then compare. It would then be useful to see if these features are really overrepresented in the rearrangement breakpoint sequences relative to non-associated real biological sequences. What about cruciforms in these regions and in the overall KSHV genome?

***The rationale, as was stated in the Results section of the original manuscript, is that “*Chromosomal rearrangement breakpoints are prone to cluster at fragile regions, typically sequences that form non-B DNA structures”. *We therefore addressed the hypothesis that non-B DNA could be contributing to rearrangement breakpoints appearing to cluster in certain regions of the KSHV genome. We restate this rationale in the* Discussion *(Lines 542 – 544).***

***On the second point, the ‘control random’ dataset was indeed generated by picking random 500-bp windows along the KSHV genome, which was detailed in the Methods section. We have added ‘*(referred to as “control permuted” in figures)’ *to that sentence to make more explicit what the controls referred to (Lines 171-172).***

***As stated in original text, no cruciform structures were predicted within the KSHV genome sequences evaluated (lines 340-341 in the revised manuscript).***

6) In addition, more careful editing and attention to details will be needed. The errors made it difficult for this reviewer and the readers to follow. For example:

(i) Table numbers seem to be incorrect throughout the result section. Table 2 should be 1, Table 3 should be 2 etc. There should be only 3 tables instead of Table 4 as referred to in the result section.

***We apologize for these obvious errors (there are, however, 4 tables in the Results section).***

(ii) There were 28 supplementary graphs as opposed to 31 mentioned in the manuscript.

***Again, we apologize. We have now added the 3 missing graphs, which are from breakpoints identified on the BCBL1 KSHV genome by other labs (see Table S2).***

(iii) Fig. S2 does not match its caption.

***We did not find an error in this caption.***

(iv) There were acronyms not described in the order of their mention throughout the manuscript;

***We are not sure which acronyms were not described when first mentioned. Many were defined in the introduction.***

(v) There were typos, even in the abstract.

***We are not sure which typos the reviewer is referring to, including those in the abstract. However, we have gone through to try to carefully identify and correct typos that we found.***

**Reviewer #2:** In this very interesting manuscript, Santiago and colleagues present the so far largest set of full-length genomic KSHV sequences obtained from KS (Kaposi Sarcoma) tumors. Their findings highlight that rearrangements and duplications of parts of the KSHV genome can occur in tumors and differ between multiple tumors observed in the same patient. They also catalogue missense mutations that occur in two KSHV genes, K4.2 and K8.1, and provide evidence that these arise during the development of individual KS lesions. Furthermore they attempt to correlate the presence of certain mutations with the development of KS disease.

The strength of this manuscript lies in the large scale and systematic analysis of KSHV genomic rearrangements and mutations. Their findings extend previous findings made in cell lines to tumors harvested from patients and thereby illustrate the biological relevance of some of the KSHV genome alterations reported in their manuscript. A weakness is that they attempt multiple statistical comparisons of individual KSHV genomic rearrangements or mutations in individual genes with tumor size, disease outcome etc., without correcting for multiple statistical comparisons.

***Addressed in detail in Part II below***

Reviewer #3: The authors present a comprehensive analysis of genomic features of Kaposi Sarcoma-associated Herpesvirus (KSHV) genomes that have been isolated from tumors. This analysis includes data from a prior publication of an initial 12 viral genomes from the same dataset. Those data are now supplemented by an additional 20 viral genomes. The introduction is superbly written, with an excellent coverage of the prior relevant literature. The analyses are thorough and clear, and the patterns observed will no doubt move the field forward. Minor areas of clarification noted below would improve this already-excellent manuscript.

***Addressed in detail in Part II below***

**Part II – Major Issues: Key Experiments Required for Acceptance**

**Reviewer #1:** Please see above.

**Reviewer #2:** 1. Figure 2: the authors should include a figure or table that lists the exact breakpoints of the amplified genomic regions, shown schematically in figure 3, that they found by Sanger sequencing of PCR products. Figure 3 does not allow to deduce these exact breakpoints.

***Each breakpoint mapped is now specified in Table S2 in the new column labelled “Breakpoint coordinate”.***

2. The multiple statistical comparisons of KSHV genomic features (amplifications, duplications, point mutations, protein truncations) with disease progression, tumor stage, etc. were done without correcting for multiple comparisons. While probably strictly not appropriate, such an approach can serve to tentatively identify genomic features that might influence disease characteristics and that could be followed up in later studies. The authors must therefore stress the tentative and explorative nature of these analyses in the abstract. They should also comment (in the discussion) on the fact that most of their KS tumors were from HIV-positive patients and that the strong effect that active HIV replication has on KS development would likely override any weak association that could exist between alterations in the KSHV genome and disease characteristics. It would be of interest to conduct a similar study in patients with HIV-negative endemic KS (in Africa) or classic KS (in Southern Europe).

***We agree and have tried to make exploratory/tentative nature of the statistical analyses cleared. We modified the abstract to include “*Exploratory evaluations of viral mutations with clinical and tumor traits were conducted by logistic regression without multiple test corrections. K5-K6 over-coverage and K8.1 inactivation were tentatively correlated” *and in the author summary: “*In an exploratory analysis these mutations were found more in tumors of nodular than macular morphotype and** **in individuals with fewer lesions.” *We added the following sentence to the* Discussion *as recommended:* “However,** **since most study participants were HIV-positive, the strong effect that active HIV replication has on KS development would likely override any weak association that could exist between KSHV genome alterations and disease characteristics. A similar study on adults with HIV-negative endemic and classic KS may strengthen this association.*” (Lines 617 – 620)***

3. Figure S5: the two groups analyzed here (WT miRK10, mutated miRK10) are of very different size, which could complicate the Kaplan-Meier analysis shown here. The Kaplan Meier curve yielded a p value of 0.038 (figure S5), whereas the Cox regression analysis presented in table S9 came to a p value of 0.053, which is also mentioned and discussed in the text. The authors should decide which analysis they want to include (probably the Cox regression) and omit the other analysis.

***We omitted the Kaplan-Meier analysis on survival rates from the paper, as recommended.***

Reviewer #3: - Figure S3 provides a valuable overview of the present study set of viral genomes and how these relate to other published sequences. The authors should consider moving this data into the main manuscript and integrating the data from Figure 6C onto this SplitsTree. Regardless of where it appears, please highlight on the SplitsTree the 65 viral genomes analyzed in this study. Otherwise the similarly named “UG..” strains are hard to distinguish from the present study’s “U..” strains.

***We integrated the phylogenetic network and the original Fig 6C into one new Fig 7. Genomes sequenced in this study were boxed.***

- Following on the above, Figure S3 clearly highlights the major clades within the KSHV tree. As illustrated in Figure 6C & Table S4, the clades do not match perfectly with the three versions of K4.2 variants or K11.2 repeats. Do any of the other variants found in multiple individuals in the present study (e.g. K8.1 or K5-K6 expansions) show any segregation on the SplitsTree? While these variants can clearly arise independently and de novo (e.g., Figure 3), there may still be an interaction with the genetic background of certain clade(s), such that these mutations are more likely to occur in one type of strain background(s) than another.

***K8.1 inactivation and K5-K6 overrepresentation data are only available for 22 specimens shown on the tree so we do not think that labelling of these is appropriate. We do not have data on the other genomes (truncated K8.1 sequences in others may have been ‘finished’ with intact K8.1 sequences, as annotated in strain GK18). This and the fact that the K4.2 and K11.2 variants do not appear to segregate with the major clades is noted in the Results section (Lines 439 - 441).***

- There are several opportunities to better integrate data across multiple figures or analyses. For example, Figure 1 has the only full-genome diagram in the manuscript. This makes it a valuable resource to refer to throughout the paper, but at present it only highlights genes of interest to Figure 1. Please consider highlighting additional genes or regions of interest from later figures on here as well (in a different color or row if need be)? E.g. K8.1, mir-K10, K4.2, K11.2, K5, K6, etc.

***Each of the genes mentioned in the paper are now shown in Figure 1A.***

- Table 1. Figure 1 and section starting on line 218 – can the authors please clarify the degree to which ddPCR and genome-sequencing did or did not agree, with regard to over-coverage of the region around IR1? The table of KSHV genome characteristics shows the ddPCR estimation of viral genomes with over-coverage around IR1 (column 7). For most of the “bolded” ratios, Figure 1B shows a good match in over-coverage by genome-sequencing to these ddPCR estimations. However the matchups do not work for viral genomes U215-D, U156-D, or U108-B – can the authors comment on why this might be, or which technique they feel is more accurate? Other samples from their prior study (ref [30]) are bolded in Table 1 but not shown in Figure 1B – how did those genomic-coverage plots match up with ddPCR estimations of duplications? These data may suggest which method(s) should be used in future studies, so this comparison is relevant to explore.

***We indicated the samples with relatively uniform ddPCR counts but which had over-coverage upon whole genome sequencing in original line 260. We have now added a sentence after that: “*This was due to the amplicons used in ddPCR being just outside the regions of over-coverage.*” (Lines 266-267)***

***As it is now clear that overrepresentation is centered on the K5-K6 region, and that “the ddPCR screen quantified genomic segments outside the minimal high coverage region” (original line 482), we added:* “****It would be useful to directly quantify via a targeted amplification approach such as ddPCR to compare K5/K6 gene copy numbers to the rest of the KSHV genome when looking for this phenomenon in the future” *(Lines 501 – 503).***

- Lines 493-498 – Can the authors speculate or point to any data on whether or not the K5-K6 over-coverage areas – and their rearrangements – might indicate that the tumors contain sub-genomic fragments containing just these regions with IR? If so, is this an in vivo correlate of defective interfering (DI) genomes or particles (DIPs), aka defective viral genomes (DVGs)? While it may well be beyond the scope of this paper to do so, is there sufficient viral DNA that methods such as RFLPs or Southern blot(s) could be used to help figure this out?

***We added to the discussion some comments on the role possible defective interfering particles of KSHV may have* in vivo*:* “Defective or deleted viral genomes in primary tumors have been reported previously for gamma-herpesviruses EBV [66,67] and KSHV [49], although their role in a natural infection is unclear.” *(Lines 529-531)***

***We appreciate the suggestion of using RFLP or Southern Blot for assessing defective viral genomes and have added that to the Discussion:* “Methods such as RFLP or Southern Blot could be used to determine whether the elevated K5-K6 genome fragments detected here exist as defective viral genomes.” *(Lines 533 – 535)***

**Part III – Minor Issues: Editorial and Data Presentation Modifications**

**Reviewer #1:**

(i) Table numbers seem to be incorrect throughout the result section. Table 2 should be 1, Table 3 should be 2 etc. There should be only 3 tables instead of Table 4 as referred to in the result section.

(ii) There were 28 supplementary graphs as opposed to 31 mentioned in the manuscript.

(iii) Fig. S2 does not match its caption.

(iv) There were acronyms not described in the order of their mention throughout the manuscript;

(v) There were typos, even in the abstract.

***All the errors we found have been addressed and discussed above.***

**Reviewer #2:**

1. Lines 246, 257, 261, 298, 420: 'Table 2' should probably be 'Table 1'.

***Table number mismatches have been fixed.***

2. Figure 5A: the meaning of the color code used for the vertical lines in the boxes denoting individual ORFs should be explained in the legend

***We added the color code of the nucleotide differences in the legend.***

3. Figure 5B: for sample UO20-E, in the line 'Translation', there should be an 'H' to indicate the coding change in the protein sequence (as for samples U004-D, 156-D).

***Because the start codon ATG was abrogated by the deletion at U020-E, no corresponding amino acid translations were written below. This is now noted in the figure legend.***

4. Line 461: the Kaplan Meier curve for the impact of the mirK10 mutation on disease progression is shown in figure S5, not S4;

***All results from Kaplan-Meier analysis on survival has been omitted in favor of the Cox regression data.***

5. lines 613ff: The legends for figures S2-S6 are either not corresponding to the individual supplementary figures of are missing.

**The revised manuscript has only 3 Supplementary figures and each legend is present in the manuscript file.**

**Reviewer #3:** Minor fixes:

- Tables & Supplemental Figures are mis-numbered and need to be updated. There are two Table 1’s, and subsequent numbering of Tables (though not their in-text references) are off. For the Supplemental Figures, Fig S1B should be Fig S2 and so on.

***The Table numbers have been fixed. We think Figs S1A – C should be kept together as they refer to each other.***

- For Figure 2 and Figure S1, is it possible to provide a diagram of the inferred rearrangement, to complement the canonical genome image and the primers? This would help to visualize how the bands match the “Sequence Interpretations” in the embedded figure-tables.

***Unfortunately, multiple interpretations on how the K5-K6 region were re-arranged can be derived from the data, precluding the requested diagram. They could have been duplicated in place, duplicated then inverted, or both. As noted in the manuscript, we could not sequence through the GC-rich regions with limited samples to assess these possibilities.***

- Line 121 – how many cycles of PCR enrichment at this step?

***That information is in the following sentence – 5 cycles. We modified the next sentence to make this clearer (Line 122).***

- Lines 149-152 – Is there any reason why read data is aligned to both the human and KSHV genomes for LUMPY analysis (breakpoint detection)? If so, it would be good to add a comment on this aspect for other researchers to consider.

***This was to look for any breakpoint joining KSHV sequences and human sequences together, indicative of an integration. No such breakpoint was found in this study, and we added this statement in the* Results *and* Discussion *sections (Lines 274 – 276 and 521 - 523).***

- Line 206 – any reason for the strong male bias in the study population? Any expected impact of this on the correlations observed (e.g. with disease manifestation(s))?

***Males constituted the majority of those presenting with KS at the Uganda Cancer Institute, which is also reflective of the known gender bias for the KSHV seroprevalence in sub-Saharan Africa.***

- Lines 205-209 – this part is quite short. It would be helpful to summarize here how the 67 tumor biopsies relate to the 29 individuals, e.g. how many people had multiple biopsies, and how many were single-sample only? How well does this availability map to the viral genomes in Table 2?

***We expanded this paragraph to add the suggested information (lines 207-209).***

- Table 1 – for the first numeric column, it’s not easy to distinguish which are “N” and which are “median”. Can these be indicated better?

***We italicized the rows where the median value is given.***

- Figure 1B – please add dash to sample names (e.g. U219-D instead of U219D) to sync with the text and table

***We relabeled as requested.***

- Figure 3 – please fix yellow and white text labels on the genome diagram; they are hard to read at present.

***We increased the sharpness and decreased the contrast of this image to make the labels stand out more.***

- Table 1, Table S4, Figure 6 – please synchronize the naming of the K4.2 length variants to be the same across text, figure, and tables. At present, Table S4 refers to Truncation A or B, while Table 1 gives only the length, and the Figure offers both options.

***We removed all references to the truncated forms as Truncated A or B.***

- Line 368 – should “intra”-host here be “inter” instead?

***Intra-here is correct; no other KSHV genes had as many intra-host mutations detected as with K8.1***

- Figure 5 – using gray bars for both the DNA (genome) and protein (translation) lines here makes the figure hard to read. Recommend changing one or the other, and then matching across other figures (e.g. in Figure 6, translations are gray bars, and in Figure 3, high-coverage areas are also gray bars).

***We changed representation of the translation in Figure 5 to blue bars in the revised manuscript.***

- Table 3 (supposed to be 4) on “Coding sequence mutations” – the footnote here is critical to the interpretation of the table and should be integrated into the table’s title.

***We implemented this recommendation***

- Figure 6C – was this tree made using full-length genomes, or just the K4.2 and K11 regions?

***Full-length genomes; this is noted in the figure legend.***

- Figure 7 – please add explanation on arrows to the figure legend

***We added in the now Fig 8 that the arrows* *indicate that the plotted range extends beyond the scale bar below.***

**Do you want your identity to be public for this peer review? For information about this choice, including consent withdrawal, please see our Privacy Policy.**

Reviewer #1: No

Reviewer #2: Yes: Thomas F. Schulz

Reviewer #3: No
