# Supplementary material for: Genomic changes in Kaposi Sarcoma-associated Herpesvirus and their clinical correlates
Source: PLoS Pathog. 2022 Nov 28;18(11):e1010524. doi: 10.1371/journal.ppat.1010524 (PMC9731496; doi:10.1371/journal.ppat.1010524)
Supplement: Attachment — Submitted filename: 2ndRound_Response to Reviewers - KSHV WGS_01Nov.docx [file ppat.1010524.s016.docx]

***Responses to Reviewer's Questions are in bold italics. Noted changes in the manuscript are highlighted.***

***We appreciate that there were very few issues with our revised submission. Only 2 issues were raised, both by Reviewer #1.***

A) ***The following is the initial critique and response to Reviewer #1:***

“3) The informatics workflow of the study was very informative, yet they could be more detailed and supported, based on the numerous tools that were used, and the fact that the KSHV genomes were de novo assembled.

***We referred to our earlier paper for the bioinformatics pipeline and tools used to generate the KSHV genomes* de novo *and also detailed any new methods. We are not sure what else would be useful without being repetitive.”***

***Reviewer #1 commented on the revision as follows (underline added to emphasize request for revision):***

Reviewer #1: The reviewers have modified the manuscript to try to address most of the concerns. They have also addressed the specific informatics comments on their control randomized sequences for comparison to the tumor genome sequences. However, it is still not fully transparent on their data analysis. Majority of their highlighted sections up until Results were unchanged, just emphasized those for the reviewers. They seem to have fixed the captions and table legends and the number of figures etc.

***In an effort to be more informative than simply referring to our previous publication, in the 2^nd^ revision of the manuscript we detailed more steps between de novo assembly and the final finished genome. We explained more how tools were used in the analysis for integration and structural variation, and we provided greater elaboration on the analysis of non-B DNA structures.***

B) “they need to cite Geneious Prime, and mention the version they are using with a URL”.

***This has now been added according to the Geneious company’s instructions.***
